# Perceived financial well-being and its association with frontostriatal functional connectivity, real-life anticipatory experiences, and everyday happiness

**DOI:** 10.1038/s41598-023-44001-0

**Published:** 2023-10-31

**Authors:** Won-Gyo Shin, Mina Jyung, Incheol Choi, Sunhae Sul

**Affiliations:** 1 Social Neuroscience Laboratory, Department of Psychology, Pusan National University, 2 Busandaehak-ro 63beon-gil, Geumjeong-gu, Busan, 46241 Republic of Korea; 2 Department of Psychology, Seoul National University, Seoul, Republic of Korea

**Keywords:** Neuroscience, Psychology, Human behaviour

## Abstract

Perceived financial well-being (FWB) is an important aspect of life that can affect one’s attitude toward future experiences and happiness. However, the relationship between FWB, anticipatory experiences, and happiness, and the brain’s functional architecture underlying this relationship remain unknown. Here, we combined an experience sampling method, multilevel modeling, and functional neuroimaging to identify the neural correlates of FWB and their associations with real-world anticipatory experiences and everyday happiness. Behaviorally, we found that individuals with greater FWB felt more positive and more interested when they expected positive events to occur, which in turn resulted in increased everyday happiness. Furthermore, the level of FWB was significantly associated with the strength of functional connectivity (FC) between the nucleus accumbens (NAc) and ventromedial prefrontal cortex (vmPFC) and the local coherence within the vmPFC. The frontostriatal FC and local coherence within the vmPFC were further predictive of everyday happiness via the anticipatory response involving interestedness during positive expectations. Our findings suggest that individual differences in FWB could be reflected in the functional architecture of brain’s reward system that may contribute to shaping positive anticipatory experiences and happiness in daily life.

## Introduction

Perceptions of one’s financial situation (i.e., perceived financial well-being; FWB) are an important aspect of individuals’ lives that can have a broad impact on happiness^1–5^. A possible mediator that links FWB and happiness may be positive anticipatory experiences. When expecting a positive event, an individual who perceives her financial situation as stable and secure would be likely to experience more positive emotional and motivational states and to fully savor upcoming positive events, compared to an individual who worries about her financial situation. Such differences in positive anticipatory experiences would ultimately influence a person’s overall well-being. In the present study, we examine how perceived FWB is associated with real-world positive anticipatory experiences and everyday happiness and how the brain’s functional architecture supports this relationship.

Recent evidence shows that perceived FWB explains substantial variance in happiness^6,7^. The effect of perceived FWB on overall well-being is comparable to the combined effect of other important life domains such as physical health, personal relationships, and job satisfaction^8^. Despite growing evidence of the relationship between FWB and happiness, the specific mechanisms through which FWB influences happiness remain unexplored. With a novel approach that combines an experience sampling method (ESM) and resting-state functional magnetic resonance imaging (rs-fMRI), we systematically examined how FWB influences real-life emotional and motivational states and everyday happiness. We propose positive anticipatory experiences as a possible psychological mechanism linking FWB to happiness.

Individuals with higher perceived FWB expect to have more resources and options, show greater flexibility in their decision-making, and experience reduced financial stress^9,10^. This can allow them to plan and prepare for upcoming positive events with a greater sense of control and empowerment in their ability to pursue their interests and make choices, without being constrained by financial concerns. Also, without financial stress, they may have more flexibility to explore a broader range of options and choose activities that truly resonate with their interests and preferences. This expanded array of opportunities can help them experience more positive emotional and motivational states (i.e., interestedness) when expecting desirable future events. Further, given that positive expectations and positive affect are key to happiness^11–13^, the relationship between perceived FWB and happiness may be mediated by positive anticipatory experiences. Considering the suggested dyadic correlations between FWB, positive anticipatory experiences, and happiness, we aim to triangulate how the relationship between FWB and happiness is mediated by positive anticipatory experiences. We specifically focused on positive experiences in this study, based on literature that suggests relatively greater variability in responses to negative FWB due to individual differences in psychological factors including adaptive coping strategies, self-efficacy, and perceived control^14–16^. Thus, we did not assume any specific relationships between FWB and negative anticipatory experiences, and instead included negative expectations in our analyses for exploratory purposes.

Importantly, we adopted rs-fMRI to further investigate the functional neural architecture supporting this relationship, which has yet to be explored. Our neuroimaging approach is grounded in literature suggesting that FWB can be tightly linked to psychological states independently of external influences. For example, poverty induces stress and negative affect, which, in turn, limit cognitive ability^17,18^, and financial stress manifests itself through mental symptoms of depression and anxiety^19^. It is therefore possible that perceptions of one’s financial situation may be associated with the functional neural patterns reflecting individual differences in the psychological states. Perceptions of sufficient financial resources and the concomitant positive affect can help individuals to pursue and engage in rewarding experiences. Experiences of such enjoyable events may be associated with better communications between or within reward-related regions. Such functional connectivity (FC) can also help better integrate reward values of financial outcomes with positive affect. Moreover, a positive sense of one’s financial situation is rewarding in and of itself.

A large body of neuroimaging research has shown that the ventral striatum, particularly the nucleus accumbens (NAc), the medial prefrontal cortex (mPFC), and broader dopaminergic brain structures mediate reward-related processes across different domains^20–24^. Of particular relevance, elevated activities in the ventral striatum and mPFC were observed in response to monetary rewards or gains^25–28^. The ventral part of the mPFC (vmPFC) plays a critical role in representing subjective value^29^ and generating affective meaning^30,31^. Given that perceptions of one’s financial situation are closely linked to appraisal or representation of the subjective value of financial rewards followed by positive affect, it is possible that perceived FWB would be associated with the FC strength between or within the NAc and vmPFC. Furthermore, vmPFC is known to be involved in representing possible future episodes and signaling affective value of the imagined episodes^32–34^. Therefore, we expected that the FC strength involving the vmPFC would also subserve anticipatory affective experiences when expecting positive events, which may influence one’s happiness.

**Figure 1:**
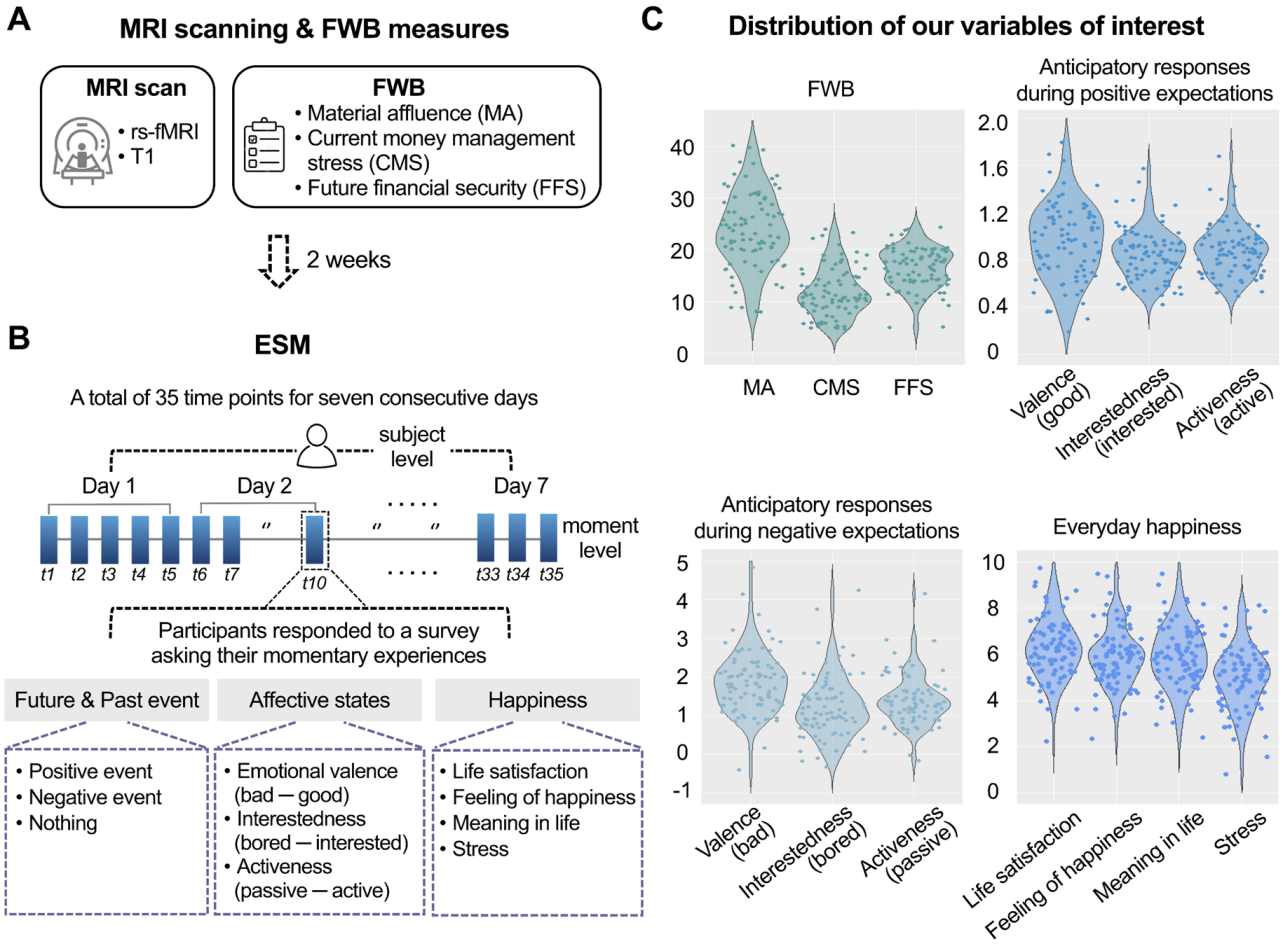
Study design and main behavioral variables. Eighty-seven healthy young adults participated in the ESM survey two weeks after completing the questionnaire of perceived FWB, including MA, CMS, and FFS, and MRI scanning consisting of anatomical T1 and rs-fMRI sessions (**A**). Five times a day for seven consecutive days, the participants were asked to report their real-world experiences, including the presence of positive or negative expectations, three domains of current affective states, and four domains of momentary happiness reflecting both subjective and psychological well-being (**B**). The average response was 30.8 times out of 35 surveys for a week, yielding a response rate of 88.01%. The total time points included in the behavioral analyses were 2680. The distributions of the major behavioral variables of our interest are presented (**C**). The degree to which participants’ affective states were influenced by the presence of upcoming positive or negative events was quantified with linear mixed-effects models. Higher scores in anticipatory responses during positive expectations indicate greater positive responses to positive future events in the measures of emotional valence (i.e., greater positive feeling), interestedness (i.e., greater interestedness), and activeness (i.e., greater activeness). In contrast, higher anticipatory response scores during negative expectations indicate greater negative responses to negative future events in the measures of emotional valence (i.e., negative feeling), interestedness (i.e., greater boredom), and activeness (i.e., greater passiveness). *ESM* experience sampling method, *FWB* financial well-being, *rs-fMRI* resting-state functional magnetic resonance imaging.

To test our ideas empirically, we leverage ESM^35^. For seven consecutive days, participants were asked to report their real-world experiences five times a day, including the presence of positive or negative expectations, current affective states, and experienced happiness (see Fig. 1 and Table S1). We measured participants’ momentary affective states using three items: emotional valence, interestedness, and activeness. As mentioned, the significance of FWB lies in its close association with access to resources and opportunities, which can influence not only general positive or negative feelings but also affective states associated with motivational processes involving engagement and initiative when expecting future events. We included interest and activeness because interest is not only a positive affective state^36^ but a source of intrinsic motivation^37,38^, and activeness is associated with a goal-driven process to achieve desired future outcomes^39^. These measures were later used to compute individuals’ anticipatory experiences, namely anticipatory responses involving emotional valence, interestedness, and activeness. In particular, the extent to which one’s affective states were influenced by the presence of positive expectation was quantified with a linear mixed-effect model. To measure happiness, we used four items covering the multifaceted nature of happiness including hedonic (i.e., life satisfaction and feelings of happiness) and eudaimonic (i.e., meaning in life) aspects of happiness, as well as stress based on its significant role in one’s well-being^40^. The scores of each happiness item were averaged across the 35 timepoints to quantify each individual’s experienced happiness as an aggregated outcome of momentary experiences, while avoiding biases and inaccuracies of retrospective global evaluations^41,42^.

Perceived FWB measures included *i*) the sense of one’s own economic affluence or scarcity (i.e., material affluence, MA), *ii*) feelings of stress or worry about one’s current financial conditions (i.e., current money management stress, CMS), and *iii*) expectations of their future financial security (i.e., future financial security, FFS). The use of these separate measures was based on the literature highlighting that each dimension has unique antecedents and consequences^4,8^. Further, we investigated the neural correlates of perceived FWB and examined whether they also support anticipatory affective experiences and momentary happiness using rs-fMRI. Prior work suggests that rs-FC (i.e., temporal correlation of spontaneous neural activity among spatially distributed brain regions) is stable within individuals and can reliably and consistently capture individuals’ mental states^43,44^. Two weeks before the ESM survey, participants reported their perceived FWB and socioeconomic status, and completed the rs-fMRI scanning.

With the ESM data, we first aimed to identify the associations among FWB, positive anticipatory experiences, and momentary happiness. Then, we performed a mediation analysis to test whether the relationship between FWB and everyday happiness is mediated by the positive anticipatory experiences. We expected that greater FWB would predict greater positive anticipatory experiences, which, in turn, would contribute to greater everyday happiness. We then employed seed-based FC and voxel-wise local FC analyses to explore whether FWB is associated with (i) the strength of FC between the NAc and the vmPFC and (ii) local coherence within those reward-related regions. Finally, we explored whether and how FCs associated with FWB are also correlated with the positive anticipatory experiences and everyday happiness.

## Results

### FWB predicts anticipatory responses during positive expectations

Prior to addressing relationships between FWB and anticipatory responses when expecting upcoming events, we first examined whether FWB measures would be associated with the rates of positive or negative expectations. Any measures of FWB were not associated with the rate of positive expectations (MA: *B* = 0.004, *t* = 1.020, *p* = 0.311; CMS: *B* =  − 0.009, *t* =  − 1.540, *p* = 0.127; FFS: *B* = 0.009, *t* = 1.223, *p* = 0.225; Table S2), while FFS negatively was correlated with the rate of negative expectations (*B* =  − 0.013, *t* =  − 3.474, *p* = 0.001; see Table S2 for the details and results of MA and CMS).

**Figure 2:**
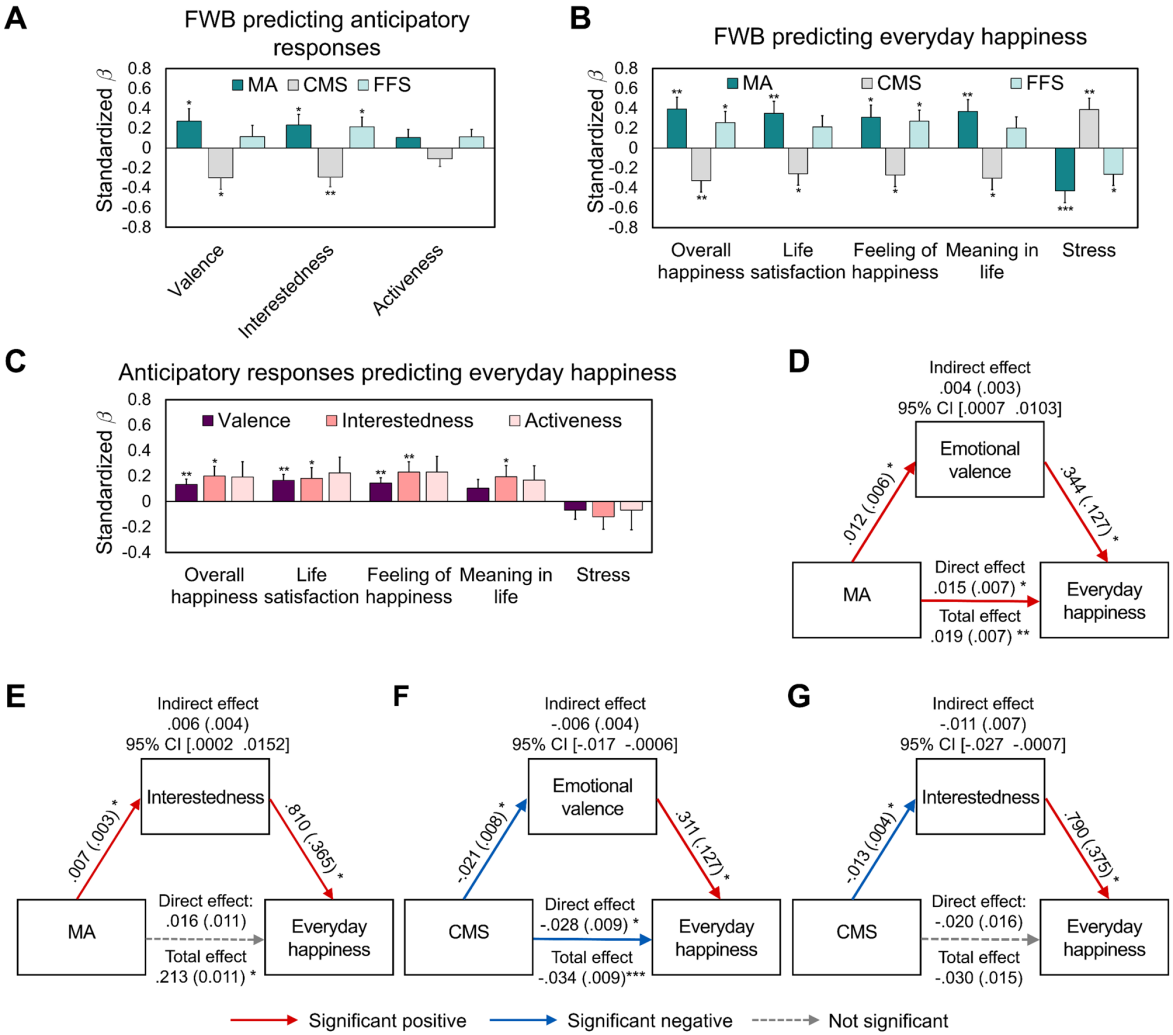
The behavioral relationships among FWB, anticipatory responses, and everyday happiness. Perceived FWB measures, including MA and CMS, significantly predicted the anticipatory response in valence and interestedness during positive expectations. FFS was also associated with anticipatory response in interestedness during positive expectations (**A**). By contrast, the affective response in negative feelings or boredom during negative expectations were not associated with FWB (see Table S3-2). All the FWB measures also predicted the composite score of everyday happiness (**B**; see Table S4 for the results of separate domains of happiness). Furthermore, the anticipatory responses during positive expectations were predictive of the composite score of everyday happiness (**C**); see Table S5 for the results of separate domains of happiness). Based on these predictive relationships among FWB, anticipatory affective experiences, and everyday happiness, we conducted mediation analyses to better understand their integrative relationships. We found that the relationships between MA and everyday happiness were positively mediated by the anticipatory response in valence (**D**) and interestedness (**E**), whereas the relationships between CMS and everyday happiness were negatively influenced by the anticipatory response in valence (**F**) and interestedness (**G**). Path coefficients are listed for each path, with standard errors in parentheses. *CMS* current money management stress, *FFS* future financial security, *MA* material affluence, *FWB* financial well-being. *p < 0.05, **p < 0.01, ***p < 0.001.

Next, we tested our hypothesis whether FWB predicted anticipatory responses when expecting upcoming positive events. As expected, all the FWB measures were associated with the anticipatory responses in both emotional valence (MA: *B* = 0.012, *t* = 2.167, *p* = 0.033; CMS: *B* =  − 0.021,* t* =  − 2.590, *p* = 0.011) and interestedness (MA: *B* = 0.007, *t* = 2.170, *p* = 0.033; CMS: *B* =  − 0.013, *t* =  − 2.981, *p* = 0.004; FFS: *B* = 0.012, *t* = 2.248, *p* = 0.027; Fig. 2A and Table S3-1). The anticipatory response in activeness was not associated with the FWB measures (Table S3-1). For exploratory purposes, we then analyzed the relationships of the FWB measures with anticipatory responses when expecting upcoming negative events, and found no significant associations (Table S3-2).

### Both FWB and anticipatory responses predict everyday happiness

Then, we examined whether FWB and the anticipatory responses would predict everyday happiness. Multiple regression analyses showed that FWB and both anticipatory responses during positive or negative expectations were significantly associated with everyday happiness. Specifically, all the FWB measures significantly predicted the composite score of happiness (MA: *B* = 0.054, *t* = 3.306, *p* = 0.001; CMS: *B* =  − 0.069, *t* =  − 2.849, *p* = 0.006; FFS: *B* = 0.065, *t* = 2.309, *p* = 0.023; Fig. 2B and Table S4). Next, the composite score of happiness was significantly predicted by the differences in affective states including valence and interestedness during positive (valence: *B* = 0.413,* t* = 3.249, *p* = 0.002; interestedness: *B* = 0.937, *t* = 2.623, *p* = 0.010) or negative expectations (valence: *B* =  − 0.348,* t* =  − 3.357, *p* = 0.001; interestedness: *B* =  − 0.432,* t* =  − 2.825, *p* = 0.006, Fig. 2C and see Table S5 for the details and results of activeness and specific measures of happiness).

### FWB predicts everyday happiness via anticipatory responses during positive expectations

So far, we confirmed the significant associations between FWB, anticipatory responses in emotional valence and interestedness during positive expectations, and everyday happiness. We found that greater FWB was associated with positive anticipatory responses in valence and interestedness during positive expectations, while the anticipatory responses during negative expectations were not influenced by FWB. Also, everyday happiness was significantly predicted by FWB and the positive anticipatory responses in valence and interestedness.

Next, we tested our hypothesis that the relationship between FWB and everyday happiness would be mediated by anticipatory responses during positive expectations. The mediation analyses revealed significant indirect effects that FWB influenced happiness via positive anticipatory responses. Specifically, the positive association between MA and the composite score of happiness was mediated by the anticipatory responses in valence and interestedness (indirect effect via valence: *B* = 0.004, standard errors (*SE*) = 0.003, 95% CI [0.0007 0.0103], Fig. 2D; indirect effect via interestedness: *B* = 0.006, *SE* = 0.004, 95% CI [0.0002 0.0152], Fig. 2E). Similarly, the negative association between CMS and the composite score of happiness was mediated by the anticipatory responses in valence and interestedness (indirect effect via valence: *B* =  − 0.006, *SE* = 0.004, 95% CI [− 0.017 − 0.0006], Fig. 2F; indirect effect via interestedness: *B* =  − 0.011, *SE* = 0.007, 95% CI [− 0.027 − 0.0007], Fig. 2G). The positive association between FFS and the composite score of happiness was not mediated by the anticipatory responses in valence and interestedness (Fig. S1A,B). The anticipatory response in activeness did not mediate the relationship between any measures of FWB and the composite score of happiness (Fig. S1C–E). To confirm that the rs-fMRI results reliably reflected our main behavioral data, we performed all the same behavioral analyses with only 54 participants with the rs-fMRI data and found similar pattens (Tables S3*, S4*, S5*; Fig. S1*).

### FC between the NAc and the vmPFC is associated with FWB

**Figure 3:**
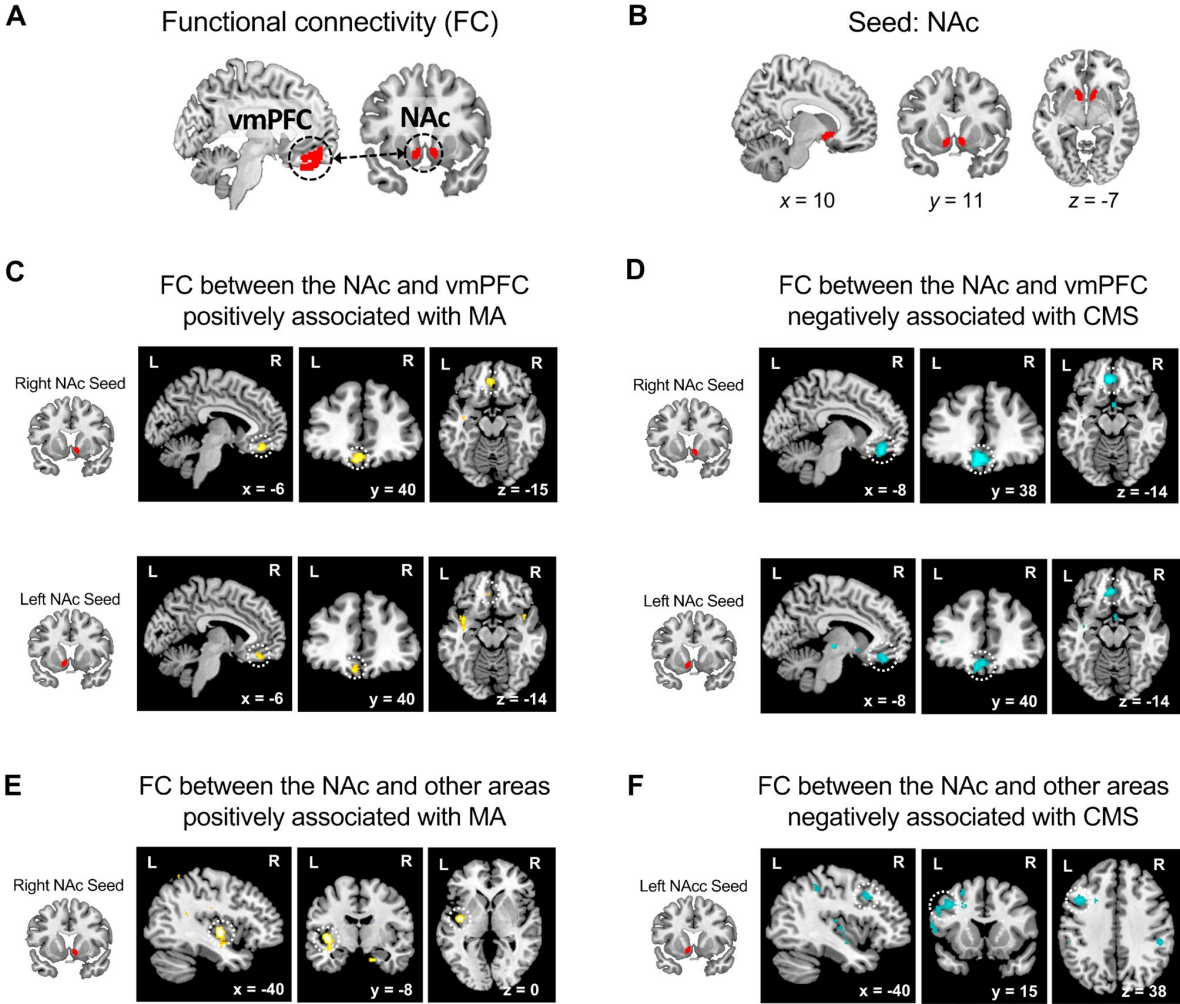
FC strength of the bilateral NAc with the vmPFC associated with FWB. We hypothesized that the FC between the NAc and the vmPFC, which are known as core regions of the brain reward system, would be associated with perceived FWB (**A**). To test this, we first created the bilateral NAc from the Harvard–Oxford Atlas (**B**) and performed seed-based FC analyses. As hypothesized, the FWB measures including MA and CMS were positively or negatively associated with the FC strength of the bilateral NAc with vmPFC ((**C**) and (**D**), respectively). These results were corrected for multiple comparisons to a significant level of SVC FWE* p* < 0.05 with an initial voxel-level threshold of* p* < 0.001. For exploratory purposes, we examined whether the FWB measures would be associated with FCs of the NAc and other brain regions without applying the ROI mask (i.e., whole-brain FWE *p* < 0.05 with the initial voxel-level threshold of *p* < 0.001). We found that the FC strength between the right NAc and the left insular was positively associated with MA, while the FC strength between the left NAc and the left IFG was negatively correlated with CMS ((**E**) and (**F**), respectively). The FCs between the NAc and the vmPFC and their association with MA and CMS remained significant in the whole-brain analyses (see Table S7). *CMS* current money management stress, *FC* functional connectivity, *FWB* financial well-being, *IFG* inferior frontal gyrus, *MA* material affluence, *NAc* nucleus accumbens, *ROI* region of interest, *SVC FWE* small volume correction family-wise error, *vmPFC* ventromedial prefrontal cortex.

After confirming the significant indirect effects of our mediation models, we conducted seed-based FC analyses to identify functional neural networks associated with FWB. Focusing on the neural networks involved in reward processing, we examined whether the FC between the NAc and the vmPFC would be correlated with FWB (Fig. 3A,B; Table S6), using the multiple regression analysis in which the FWB scores predict the FCs in the seed-based connectivity maps. Consistent with our hypothesis, MA was positively correlated with the FCs between the bilateral NAc and the vmPFC (FC between the right NAc seed and vmPFC: *x* =  − 6, *y* = 38, *z* =  − 20, *t* = 4.42; small volume correction family-wise error (SVC FWE) *p* < 0.001; FC between the left NAc seed and vmPFC: *x* =  − 6, *y* = 38, *z* =  − 18, *t* = 3.69, SVC FWE *p* = 0.016; Fig. 3C) and CMS was negatively correlated with the FCs between the bilateral NAc and the vmPFC (FC between the right NAc seed and vmPFC: *x* =  − 4, *y* = 40, *z* =  − 14,* t* =  − 6.05, SVC FWE *p* < 0.001; FC between the left NAc seed and vmPFC: *x* =  − 4, *y* = 38, *z* =  − 14, *t* =  − 4.79, SVC FWE *p* = 0.001; Fig. 3D). Lastly, unlike MA and CMS, FFS was not associated with FC strength between the NAc and the vmPFC (For exploratory seed-based connectivity analyses without a priori anatomical search volume, see Table S7 and Fig. 3E,F).

### Local coherence within the vmPFC is associated with FWB

We further performed a local coherence analysis (i.e., local FC) with the two regions of interest involved in reward processing (i.e., the NAc and the vmPFC). We found that MA was positively correlated with the local FC within the vmPFC (*x* =  − 4, *y* = 38, *z* =  − 22, *t* = 3.96; SVC FWE *p* = 0.025; Fig. 5A), while CMS was negatively correlated with the local FC within this region (*x* =  − 6, *y* = 38, *z* =  − 20, *t* =  − 4.37; SVC FWE *p* = 0.003; Fig. 5B). The local FC within the NAc was not associated with the FWB measures. Similar to the results of seed-based FC analysis, FFS was not related to the local FC within the reward-related regions.

### FCs and local coherence associated with FWB predict everyday happiness via anticipatory response involving interestedness

With the seed-based FC and local coherence results that the neural networks involving the NAc and the vmPFC were associated with FWB, we examined how these fronto-striatal functional networks contribute to everyday happiness via positive anticipatory experiences. We performed mediation analyses with the FCs associated with the FWB measures as the predictor, the anticipatory responses in valence and interestedness during positive expectations as the mediator, and the composite score of everyday happiness as the outcome variable.

**Figure 4:**
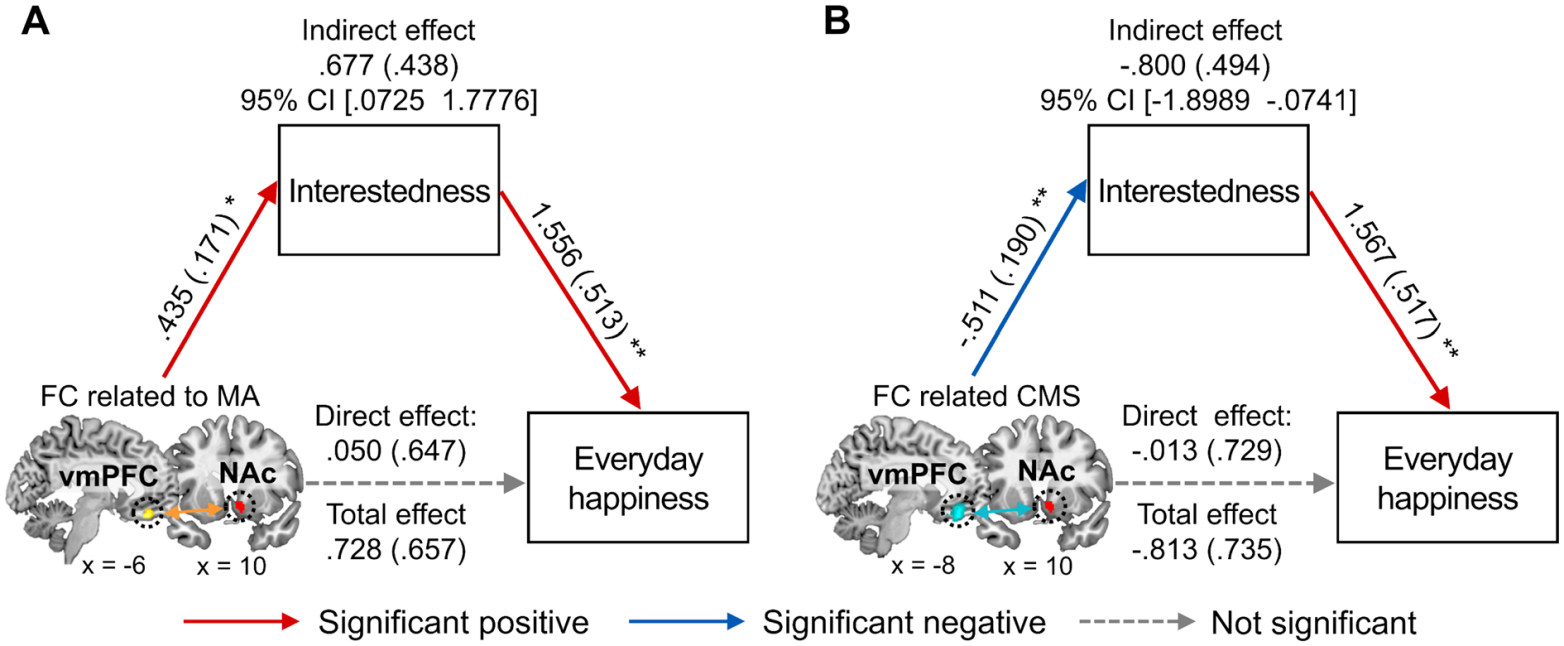
The brain-behavior relationships. Corresponding to the behavioral result, the FCs between the right NAc and the vmPFC that were associated with MA and CMS indirectly predicted everyday happiness via the anticipatory response in interestedness when expecting upcoming positive events ((**A**) and (**B**), respectively). Path coefficients are listed for each path, with standard errors in parentheses. The mediation results when entering the FCs between the left NAc and the vmPFC associated with MA and the CMS as the predictor or entering the anticipatory response in valence during the positive expectations as the mediator were presented in Figs. S2 and S3, respectively. *CMS* current money management stress, *FC* functional connectivity, *MA* material affluence, *NAc* nucleus accumbens, *vmPFC* ventromedial prefrontal cortex. *p < 0.05, **p < 0.01.

Analogous to the behavioral results, the indirect effects of the FCs on everyday happiness via positive anticipatory responses in interestedness were significant. The FCs between the right NAc and the vmPFC that were associated with MA and CMS influenced everyday happiness via the anticipatory response in interestedness (indirect effect of the FC associated with MA: *B* = 0.677, *SE* = 0.438, 95% CI [0.0725 1.7776], Fig. 4A; indirect effect of the FC associated with CMS: *B* = − 0.800, *SE* = 0.494, 95% CI [− 1.8989 − 0.0741], Fig. 4B; see Fig. S2 for the results of the FCs between the left NAc and the vmPFC). When entering the anticipatory response in valence during positive expectations as the mediator, the indirect effect was not significant (Fig. S3).

**Figure 5:**
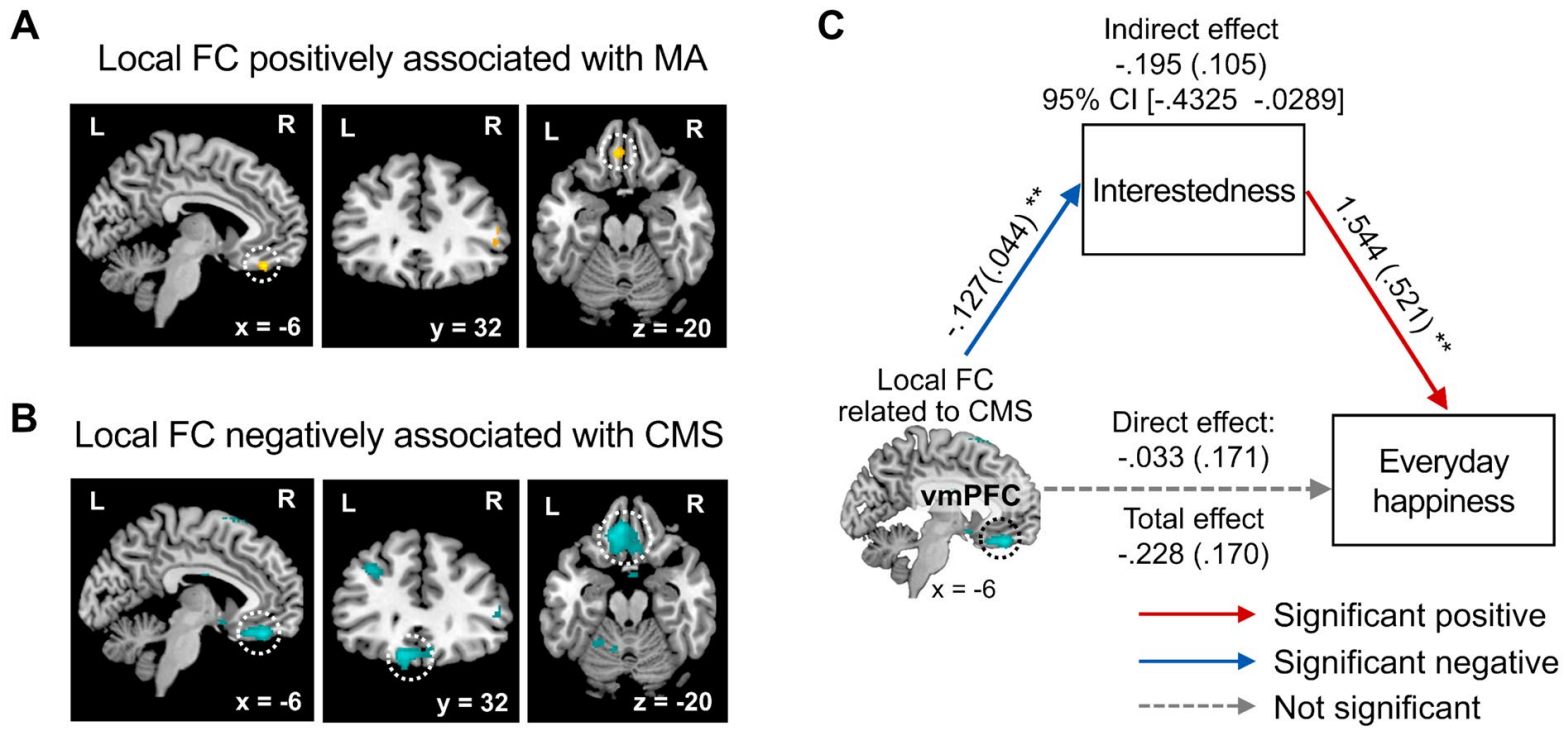
Local coherence within the vmPFC associated with FWB. The voxel-wise local FC analysis shows that MA was positively associated with local coherence within the vmPFC (**A**), whereas CMS was negatively associated with local coherence within the vmPFC (**B**). These results were corrected for multiple comparisons to a significant level of SVC FWE* p* < 0.05 with an initial voxel-level threshold of* p* < 0.001. Further, the local coherence that were associated with CMS predicted the anticipatory response in interestedness during positive expectations, which were linked to everyday happiness (**C**). Path coefficients are listed for each path, with standard errors in parentheses. The mediation results when entering the local coherence that was associated with MA as the predictor or entering the anticipatory response in valence during the positive expectations as the mediator were presented in Fig. S4. *CMS* current money management stress, *FC* functional connectivity, *MA* material affluence, *NAc* nucleus accumbens, *SVC FWE* small volume correction family-wise error, *vmPFC* ventromedial prefrontal cortex. **p < 0.01.

For the local coherence within the vmPFC associated with FWB, we found the similar mediation effects. We found a significant indirect effect of the local coherence within the vmPFC associated with CMS on everyday happiness via anticipatory response in interestedness (*B* = − 0.195, *SE* = 0.105, 95% CI [− 0.4325 − 0.0289]; Fig. 5C), whereas the indirect effect of the local coherence associated with MA via interestedness was not significant (Fig. S4). Consistent with the previous mediation analyses, the anticipatory response in valence did not significantly mediate the relationship between the local coherence within the vmPFC and happiness (Fig. S4).

## Discussion

FWB has been suggested as an important factor that contributes to one’s happiness, but the psychological mechanisms underlying this relationship—especially in terms of everyday affective experiences—remain unknown. By combining ESM, multilevel modeling, and rs-fMRI, we examined how perceived FWB is associated with the functional neural networks of reward processing to influence real-world moment-to-moment anticipatory experiences and everyday happiness. We found that FWB, especially the FWB measures for current financial affluence and stress (i.e., MA and CMS), significantly predicted everyday happiness via anticipatory responses (i.e., valence and interestedness) during positive expectations. Regarding the neural correlates, the frontostriatal FC—namely the connectivity between the NAc and the vmPFC—and the local coherence within the vmPFC were associated with MA and CMS. The frontostriatal FCs and the local coherence within the vmPFC were predictive of everyday happiness via the anticipatory response in interestedness. These findings suggest that individuals’ perceived FWB is reflected to the neural networks involved in reward processing, which may play an important role in shaping moment-to-moment positive anticipatory experiences and everyday happiness.

One of the major findings of the present study is that individuals with higher FWB were more likely to experience greater increases in emotional valence and interestedness when expecting upcoming positive events. Intriguingly, higher FWB did not predict higher frequency of positive expectations, implying that the observed effects of FWB were not the result of general positivity of happy individuals. To further test a possibility of general positivity, or a potential effect of optimism, on the relationship between FWB and the anticipatory responses, we performed additional analyses, controlling for the effect of optimism^45^ and found similar patterns of the multiple regression and mediation results (Table S3†, Fig. S1†). At least in our data, the effect of FWB seems to be associated specifically with emotional and motivational responses during the positive expectations. Previous studies have linked higher FWB with lower levels of stress and anxiety and greater control over their future in terms of financial situations^5,10^. This could provide individuals with a sense of psychological safety and help them focus on the present moment, thereby leading to greater mental energy for upcoming positive events. Therefore, individuals with high FWB may experience greater excitement and interest during expecting positive outcomes. Conversely, low FWB has been associated with being frequently preoccupied with stress and worry about finances^8,9^, which may prevent individuals from fully savoring positive anticipatory experiences.

Consistent with our expectation, we found that the relationship between the FWB measures reflecting financial affluence (i.e., MA) and financial stress (i.e., CMS) and everyday happiness was mediated by positive anticipatory responses in emotional valence and interestedness during positive expectations. Financial affluence can provide resources that allow individuals to pursue or engage in various positive activities that promote positive feelings and interest, which could contribute to everyday happiness. Conversely, financial stress or poverty can generate negative emotions^46–48^ and make it harder for individuals to enjoy upcoming positive events and to have opportunities to engage in activities or experiences that bring them joy, which can lead to lower happiness. The mediational relationship observed in our study seems to be reasonable given that positive expectations and positive affect are key to subjective well-being or happiness^11–13^.

We found asymmetric relationships of FWB with positive vs. negative expectations and with future vs. past events. First, affective responses to upcoming negative events were not associated with any FWB measures. This finding suggests the preferential influence of FWB on positive expectations. This may be because lower FWB itself is not sufficient to elicit negative affect during negative expectations, and other factors such as coping strategies and resilience can play a more important role in shaping emotional responses to negative future events. However, this should be interpreted with caution because previous research suggested that FWB can provide a buffer against negative life events^49^. Also, the rate of negative future events that participants reported in the present study was relatively lower than that of positive ones (Table S1), and thus the effect of FWB might not have been readily captured. Second, FWB did not predict emotional or motivational responses to past events (Table S8). This result provides additional evidence that our findings on the relationships of FWB with positive anticipatory experiences and happiness are not due to general positivity effect. It also suggests that FWB may be more tightly associated with preparation for future events than experience of past events. The asymmetric relationship of FWB with the future and the past merits further investigation.

Importantly, our rs-fMRI data revealed that the FWB measures reflecting financial affluence and financial stress were associated with the FC between the NAc and the vmPFC and with the local coherence within the vmPFC. The NAc and vmPFC are core regions of the brain’s reward system^20,21,29^ and constitute the fronto-striatal networks that are involved in learning and representing values of reward, including financial gains^50^. Efficient communication within the fronto-striatal network may facilitate processing of reward-related information such as feeling of being financially affluent. Also, both the fronto-striatal network and the vmPFC plays a crucial role in emotion regulation^31,51,52^. Considering that FWB can often be achieved by emotional stability and the ability to effectively regulate emotions in response to financial outcomes^53^, the increased FCs within the fronto-striatal network and the vmPFC may reflect the ability to regulate emotional responses to financial stressors. As the brain’s reward system is shaped by experiences and learning^23,54^, it may be also plausible that repeated experiences of rewarding events in financial domain help to shape stronger connectivities of the fronto-striatal reward network and within the vmPFC. As the brain-behavior relationships we found are correlational, caution should be taken when interpreting these results.

While the present study found significant relationships of the FCs with MA and CMS within the reward system, the expectation of future financial security (i.e., FFS) was not characterized by similar FC patterns. This may be because MA and CMS are associated more with immediate and tangible aspects of financial states, which can give rise to increased affective responses (i.e., positive or negative affect). By contrast, FFS is more cognitively based and captures relatively abstract and less certain aspects of FWB, and thus has less direct influence in individuals’ current affective states involving their financial situation.

Finally, the FCs associated with the FWB measures predicted everyday happiness via the anticipatory response in interestedness (but not emotional valence) during positive expectations. Given that the vmPFC is involved in episodic future thinking and processing the episode’s affective value^31–34^, our finding indicates that the FCs of the vmPFC associated with FWB can contribute to representing upcoming positive events and generating anticipatory feelings of interest. Feelings of interest may act as a form of intrinsic motivation, as they lead to goal-directed behavior and increased effort to achieve desired outcomes^36–38^, and thus could have been reliably captured in the reward-related regions. On the other hand, emotional valence measured in the present study may reflect a general state of feeling good and include a wide range of emotions^55^. Thus, the neural correlates linked to feeling good during positive expectations may be more complex and involve other regions beyond the reward and motivation systems.

The present study has limitations that should be addressed in future research. First, our data is correlational, and therefore, alternative models with different directions of influences are also possible. To seek for a psychological mechanism mediating the relationship between FWB and happiness, we designed our measurements to reflect anticipatory responses as more transient and context-dependent mechanisms and experienced happiness as an aggregated outcome of daily experiences. However, there is also a possibility that FWB increases overall happiness, which, in turn, may influence anticipatory responses. Future studies using a more stable measure of happiness, such as a questionnaire involving global retrospective evaluation of life would elucidate more fine-grained relationships among these variables. Second, the items measuring affective states and happiness may be inherently correlated because of temporal proximity and conceptual overlap. However, our operational definitions and quantifications of anticipatory responses and experienced happiness offer clear distinction between the two variables involving these items, which focused on the relationships between FWB, positive anticipatory responses, and happiness, and the underlying neural architectures. Yet, exploring common and distinct effects of positive expectations on affective states and happiness as well as more detailed relationships between the measurements could be an interesting area for future research. Third, our sample size for FC analyses was relatively small, considering a recent work highlighting the significance of very large samples for reproducible brain-behavior relationships^56^. However, our study was based on a priori hypotheses, unlike the brain-wide association studies that require very large sample sizes. Recent studies have successfully examined the relationships between neuroimaging and real-world data with a small to moderate sample sizes^57,58^. Nevertheless, future studies with larger samples would enhance the reproducibility and generalizability of our findings.

Taken together, our integrative approach provides novel, ecologically valid evidence on the neural correlates of perceived FWB and their contributions to real-world anticipatory affective experiences and everyday happiness. The functional neural architecture of the ventral fronto-striatal network, which is known to be involved in reward-related processing, seems to interact with the perception of one’s financial situation. Such perception can contribute to one’s everyday happiness via shaping positive anticipatory experiences. Future research including different demographic groups with a larger sample size and the use of both subjective and objective measures of FWB would offer a more complete view of the relationship between FWB and happiness.

## Methods

### Participants

Eighty-seven healthy adults (47 males and 40 females, mean age 21.84 ± 2.03) were recruited from a community website of Seoul National University. No participants reported any lifetime history of neurological or psychiatric illnesses or current severe depressive or anxious symptoms. A priori power analysis (G*Power^59^) assuming a moderate effect size (f^2^ = 0.15) with 80% power at an alpha level of 0.05 for linear multiple regression resulted in a desired sample size of 55. All 87 participants’ behavioral data and 56 participants’ rs-fMRI data (37 males and 19 females, mean age 22.36 ± 2.33) were available in this study, as the remaining 31 participants were scanned with different protocols for another study. Among the 56 participants with the rs-fMRI data, two were excluded due to excessive head motion (≥ 2.5 mm) or signal deviation (≥ 10% of all scans). All the participants provided written informed consent prior to their participation and were paid KRW 50,000 (approx. USD50) for their participation. All experimental protocols and methods in this study were approved by the Institutional Review Board at Seoul National University and performed in accordance with the relevant guidelines and regulations.

### fMRI data acquisition

Brain images were acquired with a Siemens 3 T Trio MRI scanner (Erlangen, Germany) using a 32-channel head coil. Small head pads were placed on both sides of the head to minimize participants’ head motion during the scanning. High-resolution, T1-weighted whole-brain anatomical scans were obtained, consisting of a magnetization-prepared rapid gradient echo sequence [repetition time (TR) = 2300 ms, echo time (TE) = 2.36 ms, field of view (FOV) = 256 × 256 mm, flip angle (FA) = 9^◦^, voxel size = 1 × 1 × 1 mm^3^, and 224 axial slices]. After the acquisition of anatomical images, rs-fMRI data were collected using a gradient echo-planar imaging pulse sequence (TR = 2000 ms, TE = 25 ms, FOV = 192 × 192 mm, FA = 80^◦^, voxel size = 3 × 3 × 3mm^3^, 38 interleaved axial slices with no gaps, and 200 volumes). During the fMRI scanning, participants were instructed to keep their eyes open and maintain the fixation.

### FWB

We assessed different aspects of perceived FWB using the following scales. First, the sense of one’s own economic affluence or scarcity was measured with the material affluence (MA) subscale from the Material Affluence Time Affluence Scale (MATAS^60^). Participants indicated their agreement with eight statements (e.g., I have had enough money to buy the things that are important to me; I have felt like I’m pretty poor) on a scale ranging from 1 (strongly disagree) to 5 (strongly agree). Second, using the Perceived Financial Well-being Scale (PFWS^8^), we measured participants’ current money management stress (CMS), which encompasses feelings of stress or worry about one’s current financial conditions and being unable to efficiently control finances. Third, from the PFWS, we also assessed participants’ expectations of their future financial security (FFS) reflecting perceptions of becoming financially secure and meeting future financial goals. Participants indicated their agreement with five statements of CMS (e.g., I am unable to enjoy life because I obsess too much about money) and five statements of FFS (e.g., I will achieve the financial goals that I have set for myself) on a scale ranging from 1 (does not describe me at all) to 5 (describes me completely). We also measured participants’ subjective socioeconomic status (SES^61^) as a control variable to examine the specific role of FWB in our analyses.

### ESM

We leveraged ESM to measure participants’ real-world experiences, including expectations of upcoming positive or negative events, occurrence of positive or negative events, within-person fluctuations in affective states, and multidimensional happiness reflecting both subjective and psychological well-being. During each of seven consecutive days, participants received a text message with a link to the ESM survey between 8:00 am and 22:59 pm. The 14-h time window was divided into five blocks of 179-min each, resulting in a total of 35 times for a week. Within each time block, a message was randomly sent with the time constraint that two consecutive messages had to be at least 30 min apart. Each ESM survey began by asking participants about their real-life experiences: (i) whether they expected a positive or negative event and (ii) whether a positive or negative event had occurred between the previous survey and the current survey. All items about affective states and happiness were rated on an 11-point Likert scale from 0 to 10. The affective states were measured by asking participants about their (i) emotional valence: how good or bad do you feel at the moment? (i.e., from 0 [very bad] to 10 [very good]) (ii) interestedness: how interested or bored do you feel at the moment? (i.e., from 0 [very bored] to 10 [very interested]) and (iii) activeness: how active or passive do you feel at the moment? (i.e., from 0 [very passive] to 10 [very active]). To assess participants’ multidimensional aspects of happiness, we asked them the following four questions: (i) how satisfied are you with your life at the moment? (i.e., from 0 [very unsatisfied] to 10 [very satisfied]) (ii) how happy do you feel at the moment? (i.e., from 0 [very unhappy] to 10 [very happy]) (iii) how meaningful do you feel your life is at the moment? (i.e., from 0 [not at all] to 10 [very much]) and (iv) how stressed out are you at the moment? (i.e., from 0 [not at all] to 10 [very much]).

### Quantification of anticipatory responses and everyday happiness

We quantified the degree to which participants’ affective states are influenced by anticipating upcoming positive or negative events (i.e., anticipatory responses) and by experiencing positive or negative past events, respectively with linear mixed-effects models using the lme4 package in R (version 4.0.4)^62^. In the models, each participant’s current affective states were entered as dependent variables and were predicted by the presence of each type of event that was coded as a dummy variable (i.e., 1 = presence, 0 = absence), respectively.

The multilevel models are specified as follows:

Level 1 model (i.e., moment level):

$$Current\, {affect}_{ij}= {\beta }_{0j}+ \left(positive \,event\right){\beta }_{1j}+ {\varepsilon }_{ij},$$

$${Current \,affect}_{ij}= {\beta }_{0j}+ \left(negative \,event\right){\beta }_{1j}+ {\varepsilon }_{ij}.$$

Level 2 model (i.e., participant level):

$${\beta }_{0j}= {\gamma }_{00}+ {\mu }_{0j},$$

$${\beta }_{1j}= {\gamma }_{10}+ {\mu }_{1j}.$$

The subscripts *i* and *j* indicate measurements at level 1 and level 2, respectively, and the parameters ${\mu }_{0}$ and ${\mu }_{1}$ denote the random effects to be estimated. The value of ${\beta }_{1j}$ was used as the extent to which individual *j*’s current affective state (i.e., valence, interestedness, or activeness) was influenced by the presence of each type of event (i.e., future positive events, future negative events, past positive events, and past negative events). ${\beta }_{0j}$ indicates the random intercept (i.e., participant *j*’s affective state when each event was absent), which was included as a covariate in all relevant analyses to rule out the effect of individuals’ baseline affect. Both ${\beta }_{1j}$ and ${\beta }_{0j}$ of each affective state were separately created to accurately capture the unique characteristics of each affective state, and thus ${\beta }_{0j}$ corresponding to a particular ${\beta }_{1j}$ was entered as a covariate when analyzing that specific affective state. The signs of the degree to which participants were influenced by negative events were reversed so that larger values indicate greater responses to the presence of negative future/past events, in the same manner as responses to the presence of positive future/past events. That is, higher scores indicate greater negative responses to negative events in the measures of emotional valence (i.e., negative feeling), interestedness (i.e., greater boredom), and activeness (i.e., greater passiveness).

Then, each participant’s everyday happiness was computed by using principal component analysis (PCA) to create a single index representing the multidimensional aspects of everyday happiness (i.e., life satisfaction, feeling of happiness, meaning in life, and stress). The PCA identified one component accounting for a total expected variance of 84.508%, and we used it as a composite score of everyday happiness. This composite score of experienced happiness served as the main dependent variable as it reflects an aggregated outcome of momentary experiences encompassing diverse aspects of life domains. In contrast, we designated the value of ${\beta }_{1j}$ as the mediator because it represents the dynamic nature of affective states triggered by specific events and can help explain the underlying mechanisms or process through which FWB influences experienced happiness. By controlling for baseline affective states (i.e., ${\beta }_{0j}$) as covariates, we aimed to isolate the unique contributions of anticipatory responses. All behavioral variables of our interest and their distributions are summarized and presented in Fig. 1 (see Table S1 for descriptive statistics of all behavioral measures).

### Statistical analyses of behavioral data

To examine the associations between the FWB, anticipatory responses in response to the anticipation of positive or negative events, and everyday happiness, we first conducted multiple linear regression analyses using IBM SPSS (version 25). Specifically, we tested the relationships *i)* between FWB and anticipatory responses, *ii)* between FWB and everyday happiness, and *iii)* between anticipatory responses and everyday happiness. For exploratory purposes, we also examined whether FWB predicted the rates of upcoming positive or negative events (i.e., frequency of positive or negative events divided total future events). Then, we conducted a mediation analysis using the PROCESS macro for SPSS^63^ with 5000 bootstrap samples to test our hypothesis that FWB would influence everyday happiness via the anticipatory responses during positive expectations. In all the analyses, age, sex, subjective SES, and the intercepts of affective states (if applicable) were controlled as covariates.

### Preprocessing of fMRI data

Preprocessing of brain images was performed using the CONN toolbox (version 20^64^, implemented in SPM12 (www.fil.ion.ucl.ac.uk/spm). Functional images were spatially realigned and unwarped to correct for between-scan motion and were slice-time corrected relative to the first slice. These images were then resampled to 2 mm isotopic voxels to the MNI space, after which they were segmented into white matter (WM), gray matter (GM), and cerebrospinal fluid (CSF) masks. The spatially normalized images were smoothed with an isotopic Gaussian kernel of 8 mm full width at half-maximum. To reduce the influence of potential outlier scans, they were detected using ART-based scrubbing tools. To remove the effects of head motion and physiological and other spurious sources of noise, the following nuisance parameters were estimated and included as regressors in the general linear model: 24 motion parameters consisting of six motion parameters, their mean-centered squares, their derivatives, and squared derivative, head motion scrubbing regressors, and five principal components extracted from both the WM and CSF masks using the CompCor method^65^. Finally, a temporal band-pass filter of 0.01 – 0.1 Hz was applied to the time series.

### Seed-based FC analysis

To identify significant associations between FWB and FC of the NAc seed region, we first generated seed-based FC maps per participant. The bilateral NAc seeds were defined from the Harvard–Oxford Atlas distributed with FSL (http://www.fmrib.ox.ac.uk/fsl/). From the individuals’ FC maps, we calculated connectivity strength using Pearson’s correlation coefficients between the timeseries of the seed region and those of all other voxels in the rest of the brain, which were then normalized by Fisher’s z transformation. To determine the regions showing a significant relationship of the bilateral NAc with FWB variables (i.e., MA, CMS, and FFS), multiple regression analyses were performed separately, while controlling for age, sex, and subjective SES. We defined a priori anatomical search volume, including the vmPFC created from the Harvard–Oxford Atlas (medial frontal cortex; MNI x, y, z = 0, 43, − 19), based on accumulated evidence demonstrating that the frontostriatal network including the NAc and the vmPFC is a core of the brain’s reward system^20,21,23,29^. To correct for multiple comparison, we ran small volume correction family-wise error (SVC FWE) *p* < 0.05 within the ROI (i.e., the cluster including the vmPFC) with initial voxel level threshold of *p* < 0.001.

### Voxel-wise local FC analysis

We further performed voxel-to-voxel analyses to investigate possible local FC at each voxel correlated with FWB measures. First, we generated local FC maps that represent a measure of local coherence at each voxel. This characterizes the average of correlation coefficients between each individual voxel and its neighboring voxels, and neighborhood was defined as a probabilistic region^66^. To identify the regions showing a significant relationship of these local FC maps with FWB measures, we performed multiple regression analyses on each measure separately, while controlling for age, sex, and subjective SES. As described above, we defined a priori anatomical search volumes including the vmPFC and the NAc created from the Harvard–Oxford Atlas, respectively. To correct for multiple comparison, SVC FWE was applied *p* < 0.05 within with the clusters including the vmPFC and the NAc with an initial voxel-level threshold of* p* < 0.001.

### Mediation analyses linking the neural data and behavioral data

Lastly, to examine whether FCs associated with FWB would predict anticipatory responses when expecting upcoming positive events leading to everyday happiness, we performed the same mediation analyses used for the behavioral data using the PROCESS macro. In the mediation models, the FC associated with each FWB measure was entered as the predictor, and the anticipatory responses involving valence, interestedness, and activeness as the mediator (tested separately), and everyday happiness as the outcome variable.

### Supplementary Information

Supplementary Information.

## Data Availability

The data and code used for the analyses are available at https://osf.io/b2ng3/. The full data set will be made available upon request due to privacy and lack of ethical approval. Further information and requests for resources should be directed to and will be fulfilled by the corresponding author, Sunhae Sul (ssul@pusan.ac.kr).
