# Supplementary material for: Perceived financial well-being and its association with frontostriatal functional connectivity, real-life anticipatory experiences, and everyday happiness
Source: Sci Rep. 2023 Oct 31;13:18739. doi: 10.1038/s41598-023-44001-0 (PMC10618479; doi:10.1038/s41598-023-44001-0)
Supplement: Supplementary file 1 — Supplementary Information. [file 41598_2023_44001_MOESM1_ESM.docx]

**Supplementary Information**

**Perceived financial well-being and its association with frontostriatal functional connectivity, real-life anticipatory experiences, and everyday happiness**

Won-Gyo Shin^1^, Mina Jyung^2^, Incheol Choi^2^, & Sunhae Sul^1,^*

^1^Department of Psychology,
Pusan National University,
Republic of Korea

^2^Department of Psychology,
Seoul National University,
Republic of Korea

*Corresponding author:
ssul@pusan.ac.kr
Social Neuroscience Laboratory
Department of Psychology
Pusan National University,
2 Busandaehakro-63beon-gil,
Geumjeong-gu, Busan, 46241, Republic of Korea
+82-51-510-2191

**Supplementary Table 1.** Descriptive statistics of all behavioral variables.

| **Variables** | **Mean (SD)** |
| --- | --- |
| Demographics |  |
| Age (N = 87) | 21.840 (2.034) |
| Male (N = 47) | 22.450 (2.348) |
| Female (N = 40) | 21.130 (1.285) |
| Subjective socioeconomic status (SES) | 6.670 (1.682) |
| FWB measures |  |
| Material affluence (MA) | 23.970 (7.279) |
| Current money management stress (CMS) | 11.89 (4.729) |
| Future financial security (FFS) | 16.48 (3.920) |
| ESM measures |  |
| Response frequency (out of 35 time points) | 30.800 (5.710) |
| Response rate | 88.013% (16.313) |
| Rate of reported events |  |
| Positive future events | 33.265 % (24.477) |
| Negative future events | 8.178 % (13.855) |
| Positive past events | 18.954 % (18.109) |
| Negative past events | 9.406 % (9.860) |
| The degree of which individuals’ affective states are influenced by upcoming positive events (i.e., affective reactivity to positive future events) | |
| Valence | .951 (.324) |
| Interestedness | .851 (.214) |
| Activeness | .875 (.212) |
| The degree of which individuals’ affective states are influenced by upcoming negative events (i.e., affective reactivity to negative future events) | |
| Valence | 1.862 (.800) |
| Interestedness | 1.192 (.835) |
| Activeness | 1.389 (.649) |
| The degree of which individuals’ affective states are influenced by positive past events (i.e., affective reactivity to positive past events) | |
| Valence | 1.372 (.055) |
| Interestedness | 1.378 (.199) |
| Activeness | 1.243 (.233) |
| The degree of which individuals’ affective states are influenced by negative past events (i.e., affective reactivity to negative past events) | |
| Valence | 1.878 (.548) |
| Interestedness | 1.118 (.560) |
| Activeness | 1.278 (.814) |
| Intercept of affective reactivity to positive future events | |
| Valence | 5.417 (.880) |
| Interestedness | 4.971 (.693) |
| Activeness | 5.155 (.747) |
| Intercept of affective reactivity to negative future events | |
| Valence | 5.900 (1.010) |
| Interestedness | 5.356 (.837) |
| Activeness | 5.580 (.885) |
| Intercept of affective reactivity to positive past events | |
| Valence | 5.465 (.931) |
| Interestedness | 5.014 (.694) |
| Activeness | 5.201 (.797) |
| Intercept of affective reactivity to positive negative events | |
| Valence | 5.899 (.989) |
| Interestedness | 5.374 (.811) |
| Activeness | 5.561 (.921) |
| Momentary everyday happiness |  |
| Composite score of happiness | 0.000 (1.000) |
| Life satisfaction | 6.240 (1.318) |
| Feeling of happiness | 5.919 (1.283) |
| Meaning in life | 5.932 (1.344) |
| Stress | 4.967 (1.378) |

*Note*. ESM, experience sampling method; FWB, financial well-being; SD, standard deviation.

**Supplementary Table 2.** Regression results of FWB as the predictor, the rate of positive or negative future events as the outcome variable, and age, sex, and subjective SES as covariates.

| **Dependent**  **Variables** | **Independent**  **Variables** | **B** | ***SE*** | ***β*** | ***t*** | ***p*** |
| --- | --- | --- | --- | --- | --- | --- |
| Rate of positive  future events | (Constant) | .849 | .362 |  | 2.346 | .021 |
|  | **MA** | **.004** | **.004** | **.127** | **1.020** | **.311** |
|  | Age | -.019 | .014 | -.160 | -1.362 | .177 |
|  | Sex | -.067 | .056 | -.138 | -1.211 | .229 |
|  | Subjective SES | -.025 | .018 | -.174 | -1.409 | .163 |
|  | (Constant) | 1.004 | .346 |  | 2.903 | .005 |
|  | **CMS** | **-.009** | **.006** | **-.182** | **-1.540** | **.127** |
|  | Age | -.017 | .014 | -.137 | -1.162 | .249 |
|  | Sex | -.064 | .055 | -.132 | -1.165 | .247 |
|  | Subjective SES | -.025 | .017 | -.175 | -1.510 | .135 |
|  | (Constant) | .801 | .368 |  | 2.175 | .033 |
|  | **FFS** | **.009** | **.007** | **.138** | **1.223** | **.225** |
|  | Age | -.020 | .014 | -.163 | -1.410 | .162 |
|  | Sex | -.064 | .056 | -.131 | -1.148 | .254 |
|  | Subjective SES | -.023 | .017 | -.156 | -1.363 | .177 |
| Rate of negative  future events | (Constant) | .060 | .206 |  | .292 | .771 |
|  | **MA** | **-.003** | **.002** | **-.157** | **-1.250** | **.215** |
|  | Age | -.001 | .008 | -.012 | -.105 | .917 |
|  | Sex | .032 | .032 | .116 | 1.014 | .314 |
|  | Subjective SES | .015 | .010 | .176 | 1.418 | .160 |
|  | (Constant) | -.048 | .196 |  | -.243 | .809 |
|  | **CMS** | **.007** | **.003** | **.224** | **1.896** | **.061** |
|  | Age | -.003 | .008 | -.040 | -.341 | .734 |
|  | Sex | .030 | .031 | .109 | .958 | .341 |
|  | Subjective SES | .015 | .010 | .177 | 1.528 | .130 |
|  | (Constant) | .222 | .198 |  | 1.120 | .266 |
|  | **FFS** | **-.013** | **.004** | **-.374** | **-3.474** | **.001** |
|  | Age | -.002 | .008 | -.034 | -.312 | .756 |
|  | Sex | .028 | .030 | .101 | .929 | .356 |
|  | Subjective SES | .017 | .009 | .211 | 1.934 | .057 |

*Note*. CMS, current money management stress; FFS, future financial security; MA, material affluence; SES, socioeconomic status.

**Supplementary Table 3-1.** Regression results of FWB as the predictor, the anticipatory responses when expecting upcoming positive events as the outcome variable, and age, sex, subjective SES, and intercept of each affective state (if applicable) as covariates.

| **Dependent**  **Variables** | **Independent**  **Variables** | **B** | ***SE*** | ***β*** | ***t*** | ***p*** |
| --- | --- | --- | --- | --- | --- | --- |
| Valence | (Constant) | -.134 | .502 |  | -.268 | .790 |
|  | **MA** | **.012** | **.006** | **.271** | **2.167** | **.033** |
|  | Age | .027 | .018 | .171 | 1.500 | .137 |
|  | Sex | .129 | .072 | .199 | 1.797 | .076 |
|  | Subjective SES | -.002 | .023 | -.010 | -.084 | .933 |
|  | Intercept | .029 | .040 | .079 | .730 | .468 |
|  | (Constant) | .246 | .496 |  | .495 | .622 |
|  | **CMS** | **-.021** | **.008** | **-.300** | **-2.590** | **.011** |
|  | Age | .032 | .018 | .198 | 1.738 | .086 |
|  | Sex | .137 | .071 | .212 | 1.934 | .057 |
|  | Subjective SES | .003 | .022 | .014 | .124 | .902 |
|  | Intercept | .033 | .039 | .090 | .859 | .393 |
|  | (Constant) | -.074 | .520 |  | -.143 | .887 |
|  | **FFS** | **.009** | **.010** | **.114** | **.989** | **.325** |
|  | Age | .022 | .018 | .141 | 1.222 | .225 |
|  | Sex | .137 | .073 | .212 | 1.867 | .066 |
|  | Subjective SES | .014 | .022 | .074 | .646 | .520 |
|  | Intercept | .041 | .041 | .112 | 1.015 | .313 |
| Interestedness | (Constant) | -.151 | .283 |  | -.532 | .596 |
|  | **MA** | **.007** | **.003** | **.232** | **2.170** | **.033** |
|  | Age | .006 | .010 | .053 | .548 | .585 |
|  | Sex | .046 | .040 | .107 | 1.137 | .259 |
|  | Subjective SES | -.013 | .013 | -.104 | -1.017 | .312 |
|  | Intercept | .157 | .029 | .511 | 5.490 | .000 |
|  | (Constant) | .091 | .278 |  | .328 | .743 |
|  | **CMS** | **-.013** | **.004** | **-.293** | **-2.981** | **.004** |
|  | Age | .009 | .010 | .085 | .884 | .379 |
|  | Sex | .050 | .039 | .118 | 1.281 | .204 |
|  | Subjective SES | -.012 | .012 | -.093 | -.982 | .329 |
|  | Intercept | .156 | .028 | .507 | 5.660 | .000 |
|  | (Constant) | -.204 | .287 |  | -.711 | .479 |
|  | **FFS** | **.012** | **.005** | **.215** | **2.248** | **.027** |
|  | Age | .004 | .010 | .041 | .433 | .666 |
|  | Sex | .051 | .040 | .120 | 1.275 | .206 |
|  | Subjective SES | -.008 | .012 | -.063 | -.660 | .511 |
|  | Intercept | .160 | .028 | .519 | 5.658 | .000 |
| Activeness | (Constant) | -.361 | .217 |  | -1.660 | .101 |
|  | **MA** | **.003** | **.002** | **.105** | **1.271** | **.207** |
|  | Age | .004 | .008 | .036 | .493 | .623 |
|  | Sex | .007 | .030 | .016 | .224 | .824 |
|  | Subjective SES | -.006 | .010 | -.047 | -.606 | .546 |
|  | Intercept | .217 | .020 | .763 | 10.720 | .000 |
|  | (Constant) | -.270 | .219 |  | -1.234 | .221 |
|  | **CMS** | **-.005** | **.003** | **-.109** | **-1.426** | **.158** |
|  | Age | .005 | .008 | .045 | .609 | .545 |
|  | Sex | .009 | .030 | .021 | .286 | .775 |
|  | Subjective SES | -.005 | .009 | -.036 | -.489 | .626 |
|  | Intercept | .218 | .020 | .770 | 11.111 | .000 |
|  | (Constant) | -.389 | .219 |  | -1.779 | .079 |
|  | **FFS** | **.006** | **.004** | **.113** | **1.516** | **.133** |
|  | Age | .003 | .008 | .033 | .452 | .652 |
|  | Sex | .009 | .030 | .022 | .310 | .757 |
|  | Subjective SES | -.004 | .009 | -.032 | -.446 | .656 |
|  | Intercept | .216 | .020 | .760 | 10.762 | .000 |

*Note*. CMS, current money management stress; FFS, future financial security; MA, material affluence; SES, socioeconomic status.

**Supplementary Table 3-2.** Regression results of FWB as the predictor, the anticipatory responses when expecting upcoming negative events as the outcome variable, and age, sex, subjective SES, and intercept of each affective state (if applicable) as covariates.

| **Dependent**  **Variables** | **Independent**  **Variables** | **B** | ***SE*** | ***β*** | ***t*** | ***p*** |
| --- | --- | --- | --- | --- | --- | --- |
| Valence | (Constant) | -3.134 | .737 |  | -4.253 | .000 |
|  | **MA** | **-.012** | **.008** | **-.106** | **-1.406** | **.164** |
|  | Age | .043 | .027 | .108 | 1.572 | .120 |
|  | Sex | .326 | .107 | .204 | 3.046 | .003 |
|  | Subjective SES | .074 | .034 | .156 | 2.152 | .034 |
|  | Intercept | .627 | .052 | .792 | 12.080 | .000 |
|  | (Constant) | -3.467 | .734 |  | -4.721 | .000 |
|  | **CMS** | **.016** | **.012** | **.092** | **1.291** | **.200** |
|  | Age | .041 | .028 | .104 | 1.484 | .142 |
|  | Sex | .319 | .107 | .200 | 2.979 | .004 |
|  | Subjective SES | .066 | .033 | .139 | 2.022 | .047 |
|  | Intercept | .621 | .051 | .784 | 12.113 | .000 |
|  | (Constant) | -3.082 | .755 |  | -4.082 | .000 |
|  | **FFS** | **-.016** | **.014** | **-.080** | **-1.189** | **.238** |
|  | Age | .045 | .027 | .116 | 1.688 | .095 |
|  | Sex | .317 | .107 | .199 | 2.959 | .004 |
|  | Subjective SES | .063 | .032 | .133 | 1.958 | .054 |
|  | Intercept | .619 | .051 | .781 | 12.092 | .000 |
| Interestedness | (Constant) | -3.982 | .704 |  | -5.657 | .000 |
|  | **MA** | **-.010** | **.008** | **-.085** | **-1.231** | **.222** |
|  | Age | .024 | .026 | .058 | .914 | .364 |
|  | Sex | .231 | .102 | .139 | 2.262 | .026 |
|  | Subjective SES | .040 | .033 | .081 | 1.224 | .225 |
|  | Intercept | .843 | .060 | .844 | 14.068 | .000 |
|  | (Constant) | -4.286 | .700 |  | -6.119 | .000 |
|  | **CMS** | **.015** | **.011** | **.083** | **1.281** | **.204** |
|  | Age | .021 | .026 | .052 | .811 | .420 |
|  | Sex | .225 | .102 | .135 | 2.208 | .030 |
|  | Subjective SES | .035 | .031 | .070 | 1.120 | .266 |
|  | Intercept | .840 | .059 | .842 | 14.163 | .000 |
|  | (Constant) | -3.978 | .722 |  | -5.512 | .000 |
|  | **FFS** | **-.011** | **.013** | **-.052** | **-.849** | **.399** |
|  | Age | .027 | .026 | .065 | 1.042 | .300 |
|  | Sex | .225 | .103 | .135 | 2.195 | .031 |
|  | Subjective SES | .030 | .031 | .060 | .963 | .338 |
|  | Intercept | .834 | .059 | .835 | 14.065 | .000 |
| Activeness | (Constant) | -1.192 | .853 |  | -1.397 | .166 |
|  | **MA** | **-.005** | **.010** | **-.056** | **-.523** | **.602** |
|  | Age | .008 | .031 | .026 | .275 | .784 |
|  | Sex | .149 | .121 | .115 | 1.227 | .223 |
|  | Subjective SES | -.010 | .039 | -.025 | -.248 | .805 |
|  | Intercept | .450 | .068 | .614 | 6.637 | .000 |
|  | (Constant) | -1.260 | .858 |  | -1.469 | .146 |
|  | **CMS** | **.000** | **.014** | **.002** | **.022** | **.983** |
|  | Age | .011 | .031 | .035 | .355 | .723 |
|  | Sex | .147 | .121 | .114 | 1.213 | .229 |
|  | Subjective SES | -.018 | .037 | -.048 | -.495 | .622 |
|  | Intercept | .441 | .067 | .601 | 6.619 | .000 |
|  | (Constant) | -1.180 | .867 |  | -1.360 | .178 |
|  | **FFS** | **-.006** | **.016** | **-.037** | **-.389** | **.699** |
|  | Age | .010 | .030 | .031 | .323 | .748 |
|  | Sex | .146 | .121 | .112 | 1.200 | .234 |
|  | Subjective SES | -.015 | .036 | -.039 | -.409 | .683 |
|  | Intercept | .446 | .067 | .609 | 6.680 | .000 |

*Note*. CMS, current money management stress; FFS, future financial security; MA, material affluence; SES, socioeconomic status.

**Supplementary Table 3-1*.** Regression results of FWB as the predictor, the anticipatory responses when expecting upcoming positive events as the outcome variable, and age, sex, subjective SES, and intercept of each affective state (if applicable) as covariates (*N* = 54).

| **Dependent**  **Variables** | **Independent**  **Variables** | **B** | ***SE*** | ***β*** | ***t*** | ***p*** |
| --- | --- | --- | --- | --- | --- | --- |
| Valence | (Constant) | -.938 | .664 |  | -1.414 | .164 |
|  | **MA** | **.016** | **.008** | **.317** | **2.070** | **.044** |
|  | Age | .042 | .021 | .278 | 1.961 | .056 |
|  | Sex | .155 | .099 | .210 | 1.565 | .124 |
|  | Subjective SES | .029 | .029 | .143 | 1.003 | .321 |
|  | Intercept | .062 | .055 | .150 | 1.115 | .271 |
|  | (Constant) | -.303 | .647 |  | -.469 | .642 |
|  | **CMS** | **-.029** | **.010** | **-.413** | **-2.849** | **.006** |
|  | Age | .048 | .021 | .321 | 2.312 | .025 |
|  | Sex | .179 | .095 | .243 | 1.882 | .066 |
|  | Subjective SES | .026 | .028 | .125 | .930 | .357 |
|  | Intercept | .056 | .053 | .137 | 1.062 | .293 |
|  | (Constant) | -.788 | .680 |  | -1.159 | .252 |
|  | **FFS** | **.013** | **.013** | **.153** | **1.014** | **.316** |
|  | Age | .035 | .022 | .234 | 1.624 | .111 |
|  | Sex | .171 | .102 | .232 | 1.682 | .099 |
|  | Subjective SES | .044 | .029 | .217 | 1.529 | .133 |
|  | Intercept | .073 | .059 | .178 | 1.246 | .219 |
| Interestedness | (Constant) | -.363 | .361 |  | -1.008 | .319 |
|  | **MA** | **.007** | **.004** | **.228** | **1.690** | **.098** |
|  | Age | .009 | .012 | .102 | .813 | .420 |
|  | Sex | .042 | .054 | .092 | .780 | .439 |
|  | Subjective SES | .010 | .016 | .076 | .598 | .553 |
|  | Intercept | .153 | .037 | .494 | 4.146 | .000 |
|  | (Constant) | -.016 | .344 |  | -.045 | .964 |
|  | **CMS** | **-.016** | **.006** | **-.371** | **-2.913** | **.005** |
|  | Age | .014 | .011 | .152 | 1.255 | .216 |
|  | Sex | .054 | .051 | .120 | 1.071 | .290 |
|  | Subjective SES | .005 | .015 | .044 | .369 | .714 |
|  | Intercept | .140 | .035 | .453 | 3.983 | .000 |
|  | (Constant) | -.345 | .360 |  | -.957 | .343 |
|  | **FFS** | **.010** | **.007** | **.202** | **1.591** | **.118** |
|  | Age | .008 | .011 | .082 | .659 | .513 |
|  | Sex | .049 | .054 | .109 | .915 | .365 |
|  | Subjective SES | .013 | .015 | .102 | .833 | .409 |
|  | Intercept | .152 | .037 | .492 | 4.097 | .000 |
| Activeness | (Constant) | -.739 | .267 |  | -2.769 | .008 |
|  | **MA** | **.001** | **.003** | **.039** | **.393** | **.696** |
|  | Age | .012 | .009 | .124 | 1.386 | .172 |
|  | Sex | .023 | .040 | .048 | .574 | .569 |
|  | Subjective SES | .018 | .012 | .134 | 1.484 | .144 |
|  | Intercept | .230 | .025 | .807 | 9.176 | .000 |
|  | (Constant) | -.663 | .274 |  | -2.424 | .019 |
|  | **CMS** | **-.004** | **.004** | **-.081** | **-.839** | **.406** |
|  | Age | .013 | .009 | .136 | 1.512 | .137 |
|  | Sex | .026 | .040 | .054 | .646 | .521 |
|  | Subjective SES | .016 | .012 | .122 | 1.390 | .171 |
|  | Intercept | .226 | .025 | .795 | 9.202 | .000 |
|  | (Constant) | -.751 | .264 |  | -2.843 | .007 |
|  | **FFS** | **.005** | **.005** | **.089** | **.946** | **.349** |
|  | Age | .012 | .008 | .125 | 1.438 | .157 |
|  | Sex | .025 | .040 | .052 | .630 | .531 |
|  | Subjective SES | .016 | .011 | .123 | 1.433 | .158 |
|  | Intercept | .224 | .025 | .786 | 8.880 | .000 |

*Note*. CMS, current money management stress; FFS, future financial security; MA, material affluence; SES, socioeconomic status.

**Supplementary Table 3-2*.** Regression results of FWB as the predictor, the anticipatory responses when expecting upcoming negative events as the outcome variable, and age, sex, subjective SES, and intercept of each affective state (if applicable) as covariates (*N* = 54).

| **Dependent**  **Variables** | **Independent**  **Variables** | **B** | ***SE*** | ***β*** | ***t*** | ***p*** |
| --- | --- | --- | --- | --- | --- | --- |
| Valence | (Constant) | -3.721 | 1.045 |  | -3.560 | .001 |
|  | **MA** | **-.013** | **.013** | **-.100** | **-.987** | **.328** |
|  | Age | .051 | .035 | .137 | 1.464 | .150 |
|  | Sex | .278 | .161 | .152 | 1.728 | .090 |
|  | Subjective SES | .057 | .048 | .112 | 1.184 | .242 |
|  | Intercept | .713 | .079 | .814 | 9.067 | .000 |
|  | (Constant) | -4.106 | 1.045 |  | -3.929 | .000 |
|  | **CMS** | **.014** | **.017** | **.080** | **.807** | **.424** |
|  | Age | .051 | .036 | .136 | 1.431 | .159 |
|  | Sex | .263 | .162 | .144 | 1.628 | .110 |
|  | Subjective SES | .052 | .047 | .102 | 1.093 | .280 |
|  | Intercept | .707 | .078 | .807 | 9.038 | .000 |
|  | (Constant) | -3.556 | 1.027 |  | -3.463 | .001 |
|  | **FFS** | **-.032** | **.019** | **-.155** | **-1.702** | **.095** |
|  | Age | .051 | .034 | .136 | 1.505 | .139 |
|  | Sex | .261 | .158 | .143 | 1.655 | .104 |
|  | Subjective SES | .060 | .045 | .119 | 1.330 | .190 |
|  | Intercept | .722 | .076 | .824 | 9.457 | .000 |
| Interestedness | (Constant) | -4.445 | 1.004 |  | -4.427 | .000 |
|  | **MA** | **-.010** | **.012** | **-.075** | **-.801** | **.427** |
|  | Age | .035 | .033 | .092 | 1.043 | .302 |
|  | Sex | .210 | .155 | .113 | 1.354 | .182 |
|  | Subjective SES | .059 | .046 | .115 | 1.280 | .207 |
|  | Intercept | .858 | .089 | .812 | 9.595 | .000 |
|  | (Constant) | -4.829 | .985 |  | -4.904 | .000 |
|  | **CMS** | **.016** | **.016** | **.091** | **.982** | **.331** |
|  | Age | .032 | .034 | .084 | .939 | .353 |
|  | Sex | .194 | .155 | .105 | 1.253 | .216 |
|  | Subjective SES | .060 | .045 | .116 | 1.317 | .194 |
|  | Intercept | .864 | .090 | .818 | 9.622 | .000 |
|  | (Constant) | -4.413 | 1.002 |  | -4.403 | .000 |
|  | **FFS** | **-.017** | **.018** | **-.080** | **-.928** | **.358** |
|  | Age | .037 | .033 | .096 | 1.113 | .271 |
|  | Sex | .199 | .155 | .107 | 1.288 | .204 |
|  | Subjective SES | .057 | .045 | .111 | 1.274 | .209 |
|  | Intercept | .858 | .089 | .812 | 9.657 | .000 |
| Activeness | (Constant) | -2.191 | 1.022 |  | -2.144 | .037 |
|  | **MA** | **-.009** | **.013** | **-.093** | **-.703** | **.485** |
|  | Age | .034 | .034 | .120 | .999 | .323 |
|  | Sex | .165 | .158 | .119 | 1.049 | .299 |
|  | Subjective SES | .057 | .047 | .147 | 1.213 | .231 |
|  | Intercept | .464 | .084 | .659 | 5.524 | .000 |
|  | (Constant) | -2.540 | 1.034 |  | -2.457 | .018 |
|  | **CMS** | **.016** | **.017** | **.116** | **.898** | **.373** |
|  | Age | .031 | .035 | .109 | .896 | .375 |
|  | Sex | .151 | .157 | .109 | .959 | .343 |
|  | Subjective SES | .058 | .046 | .150 | 1.264 | .212 |
|  | Intercept | .467 | .083 | .663 | 5.640 | .000 |
|  | (Constant) | -2.089 | 1.013 |  | -2.062 | .045 |
|  | **FFS** | **-.023** | **.019** | **-.149** | **-1.232** | **.224** |
|  | Age | .034 | .033 | .119 | 1.020 | .313 |
|  | Sex | .152 | .156 | .109 | .973 | .336 |
|  | Subjective SES | .059 | .045 | .154 | 1.330 | .190 |
|  | Intercept | .474 | .082 | .673 | 5.791 | .000 |

*Note*. CMS, current money management stress; FFS, future financial security; MA, material affluence; SES, socioeconomic status.

**Supplementary Table 3-1†.** Regression results of FWB as the predictor, the anticipatory responses when expecting upcoming positive events as the outcome variable, and age, sex, subjective SES, optimism, and intercept of each affective state (if applicable) as covariates.

| **Dependent**  **Variables** | **Independent**  **Variables** | **B** | ***SE*** | ***β*** | ***t*** | ***p*** |
| --- | --- | --- | --- | --- | --- | --- |
| Valence | (Constant) | -.029 | .597 |  | -.048 | .962 |
|  | **MA** | **.012** | **.006** | **.273** | **2.169** | **.033** |
|  | Age | .027 | .018 | .167 | 1.458 | .149 |
|  | Sex | .128 | .072 | .198 | 1.772 | .080 |
|  | Subjective SES | -.002 | .023 | -.011 | -.094 | .925 |
|  | Intercept | .029 | .040 | .078 | .713 | .478 |
|  | Optimism | -.005 | .016 | -.035 | -.331 | .741 |
|  | (Constant) | .391 | .596 |  | .657 | .513 |
|  | **CMS** | **-.021** | **.008** | **-.304** | **-2.606** | **.011** |
|  | Age | .031 | .018 | .194 | 1.692 | .095 |
|  | Sex | .136 | .071 | .210 | 1.905 | .060 |
|  | Subjective SES | .002 | .022 | .012 | .106 | .916 |
|  | Intercept | .033 | .039 | .088 | .835 | .406 |
|  | Optimism | -.007 | .016 | -.047 | -.446 | .657 |
|  | (Constant) | .035 | .612 |  | .057 | .955 |
|  | **FFS** | **.010** | **.010** | **.119** | **1.019** | **.311** |
|  | Age | .022 | .019 | .138 | 1.184 | .240 |
|  | Sex | .136 | .074 | .210 | 1.842 | .069 |
|  | Subjective SES | .014 | .022 | .072 | .626 | .533 |
|  | Intercept | .040 | .041 | .110 | .989 | .326 |
|  | Optimism | -.006 | .016 | -.037 | -.344 | .732 |
| Interestedness | (Constant) | -.193 | .336 |  | -.574 | .567 |
|  | **MA** | **.007** | **.003** | **.231** | **2.143** | **.035** |
|  | Age | .006 | .010 | .055 | .563 | .575 |
|  | Sex | .046 | .040 | .108 | 1.139 | .258 |
|  | Subjective SES | -.013 | .013 | -.103 | -1.002 | .319 |
|  | Intercept | .158 | .029 | .511 | 5.463 | .000 |
|  | Optimism | .002 | .009 | .021 | .238 | .813 |
|  | (Constant) | .074 | .333 |  | .221 | .826 |
|  | **CMS** | **-.013** | **.004** | **-.292** | **-2.944** | **.004** |
|  | Age | .009 | .010 | .086 | .884 | .380 |
|  | Sex | .050 | .039 | .118 | 1.276 | .206 |
|  | Subjective SES | -.012 | .012 | -.092 | -.972 | .334 |
|  | Intercept | .156 | .028 | .507 | 5.625 | .000 |
|  | Optimism | .001 | .009 | .009 | .099 | .921 |
|  | (Constant) | -.216 | .337 |  | -.642 | .523 |
|  | **FFS** | **.012** | **.005** | **.215** | **2.209** | **.030** |
|  | Age | .004 | .010 | .042 | .434 | .665 |
|  | Sex | .051 | .040 | .120 | 1.268 | .208 |
|  | Subjective SES | -.008 | .012 | -.062 | -.652 | .516 |
|  | Intercept | .160 | .028 | .519 | 5.621 | .000 |
|  | Optimism | .001 | .009 | .006 | .070 | .944 |
| Activeness | (Constant) | -.366 | .251 |  | -1.457 | .149 |
|  | **MA** | **.003** | **.002** | **.105** | **1.263** | **.210** |
|  | Age | .004 | .008 | .037 | .492 | .624 |
|  | Sex | .007 | .031 | .016 | .224 | .824 |
|  | Subjective SES | -.006 | .010 | -.047 | -.601 | .549 |
|  | Intercept | .217 | .020 | .763 | 10.584 | .000 |
|  | Optimism | .000 | .007 | .003 | .039 | .969 |
|  | (Constant) | -.266 | .253 |  | -1.050 | .297 |
|  | **CMS** | **-.005** | **.003** | **-.109** | **-1.416** | **.161** |
|  | Age | .005 | .008 | .045 | .601 | .550 |
|  | Sex | .009 | .031 | .020 | .283 | .778 |
|  | Subjective SES | -.005 | .009 | -.036 | -.487 | .628 |
|  | Intercept | .219 | .020 | .770 | 10.986 | .000 |
|  | Optimism | .000 | .007 | -.002 | -.032 | .975 |
|  | (Constant) | -.379 | .250 |  | -1.514 | .134 |
|  | **FFS** | **.006** | **.004** | **.113** | **1.508** | **.135** |
|  | Age | .003 | .008 | .032 | .442 | .660 |
|  | Sex | .009 | .030 | .022 | .305 | .761 |
|  | Subjective SES | -.004 | .009 | -.032 | -.447 | .656 |
|  | Intercept | .216 | .020 | .760 | 10.660 | .000 |
|  | Optimism | -.001 | .007 | -.006 | -.081 | .936 |

*Note*. CMS, current money management stress; FFS, future financial security; MA, material affluence; SES, socioeconomic status.

**Supplementary Table 3-2†.** Regression results of FWB as the predictor, the anticipatory responses when expecting upcoming negative events as the outcome variable, and age, sex, subjective SES, optimism, and intercept of each affective state (if applicable) as covariates.

| **Dependent**  **Variables** | **Independent**  **Variables** | **B** | ***SE*** | ***β*** | ***t*** | ***p*** |
| --- | --- | --- | --- | --- | --- | --- |
| Valence | (Constant) | -3.784 | .862 |  | -4.387 | .000 |
|  | **MA** | **-.012** | **.008** | **-.110** | **-1.464** | **.147** |
|  | Age | .046 | .027 | .117 | 1.698 | .093 |
|  | Sex | .332 | .106 | .208 | 3.119 | .003 |
|  | Subjective SES | .076 | .034 | .159 | 2.212 | .030 |
|  | Intercept | .626 | .052 | .790 | 12.126 | .000 |
|  | Optimism | .033 | .023 | .090 | 1.425 | .158 |
|  | (Constant) | -4.160 | .867 |  | -4.796 | .000 |
|  | **CMS** | **.017** | **.012** | **.099** | **1.408** | **.163** |
|  | Age | .044 | .027 | .111 | 1.598 | .114 |
|  | Sex | .325 | .106 | .204 | 3.053 | .003 |
|  | Subjective SES | .068 | .033 | .143 | 2.096 | .039 |
|  | Intercept | .620 | .051 | .782 | 12.175 | .000 |
|  | Optimism | .034 | .023 | .093 | 1.474 | .144 |
|  | (Constant) | -3.750 | .869 |  | -4.314 | .000 |
|  | **FFS** | **-.019** | **.014** | **-.092** | **-1.360** | **.178** |
|  | Age | .049 | .027 | .124 | 1.817 | .073 |
|  | Sex | .323 | .107 | .202 | 3.033 | .003 |
|  | Subjective SES | .066 | .032 | .138 | 2.044 | .044 |
|  | Intercept | .618 | .051 | .780 | 12.168 | .000 |
|  | Optimism | .035 | .023 | .096 | 1.515 | .134 |
| Interestedness | (Constant) | -4.296 | .831 |  | -5.173 | .000 |
|  | **MA** | **-.010** | **.008** | **-.086** | **-1.251** | **.215** |
|  | Age | .025 | .026 | .062 | .970 | .335 |
|  | Sex | .234 | .102 | .140 | 2.283 | .025 |
|  | Subjective SES | .041 | .033 | .083 | 1.244 | .217 |
|  | Intercept | .842 | .060 | .843 | 14.003 | .000 |
|  | Optimism | .016 | .022 | .042 | .717 | .475 |
|  | (Constant) | -4.634 | .834 |  | -5.554 | .000 |
|  | **CMS** | **.015** | **.012** | **.087** | **1.332** | **.187** |
|  | Age | .023 | .026 | .055 | .864 | .390 |
|  | Sex | .228 | .102 | .137 | 2.230 | .029 |
|  | Subjective SES | .036 | .031 | .073 | 1.149 | .254 |
|  | Intercept | .840 | .060 | .841 | 14.109 | .000 |
|  | Optimism | .017 | .022 | .045 | .772 | .443 |
|  | (Constant) | -4.305 | .839 |  | -5.132 | .000 |
|  | **FFS** | **-.012** | **.013** | **-.058** | **-.927** | **.357** |
|  | Age | .028 | .026 | .069 | 1.098 | .276 |
|  | Sex | .228 | .103 | .137 | 2.215 | .030 |
|  | Subjective SES | .031 | .031 | .063 | .997 | .322 |
|  | Intercept | .833 | .059 | .834 | 14.014 | .000 |
|  | Optimism | .017 | .023 | .045 | .770 | .443 |
| Activeness | (Constant) | -1.675 | .978 |  | -1.712 | .091 |
|  | **MA** | **-.005** | **.010** | **-.055** | **-.519** | **.605** |
|  | Age | .011 | .031 | .035 | .360 | .720 |
|  | Sex | .154 | .121 | .119 | 1.268 | .208 |
|  | Subjective SES | -.009 | .039 | -.022 | -.219 | .828 |
|  | Intercept | .439 | .069 | .599 | 6.394 | .000 |
|  | Optimism | .027 | .027 | .090 | 1.009 | .316 |
|  | (Constant) | -1.752 | .986 |  | -1.777 | .079 |
|  | **CMS** | **.001** | **.014** | **.007** | **.071** | **.944** |
|  | Age | .013 | .031 | .042 | .427 | .671 |
|  | Sex | .152 | .121 | .118 | 1.254 | .214 |
|  | Subjective SES | -.017 | .037 | -.043 | -.446 | .656 |
|  | Intercept | .430 | .067 | .587 | 6.390 | .000 |
|  | Optimism | .027 | .027 | .091 | 1.011 | .315 |
|  | (Constant) | -1.665 | .983 |  | -1.694 | .094 |
|  | **FFS** | **-.007** | **.016** | **-.045** | **-.474** | **.637** |
|  | Age | .012 | .031 | .038 | .401 | .689 |
|  | Sex | .150 | .121 | .116 | 1.241 | .218 |
|  | Subjective SES | -.013 | .036 | -.033 | -.352 | .726 |
|  | Intercept | .436 | .067 | .595 | 6.465 | .000 |
|  | Optimism | .028 | .027 | .094 | 1.046 | .299 |

*Note*. CMS, current money management stress; FFS, future financial security; MA, material affluence; SES, socioeconomic status.

**Supplementary Table 4.** Regression results of FWB as the predictor, momentary happiness as the outcome variable, and age, sex, and subjective SES as covariates.

| **Dependent**  **Variables** | **Independent**  **Variables** | **B** | ***SE*** | ***β*** | ***t*** | ***p*** |
| --- | --- | --- | --- | --- | --- | --- |
| Composite score  of happiness | (Constant) | .297 | 1.404 |  | .212 | .833 |
|  | **MA** | **.054** | **.016** | **.392** | **3.306** | **.001** |
|  | Age | -.052 | .055 | -.105 | -.947 | .346 |
|  | Sex | -.193 | .216 | -.097 | -.894 | .374 |
|  | Subjective SES | -.055 | .070 | -.093 | -.790 | .432 |
|  | (Constant) | 1.982 | 1.372 |  | 1.444 | .152 |
|  | **CMS** | **-.069** | **.024** | **-.327** | **-2.849** | **.006** |
|  | Age | -.046 | .056 | -.093 | -.810 | .420 |
|  | Sex | -.163 | .219 | -.082 | -.743 | .460 |
|  | Subjective SES | -.013 | .067 | -.023 | -.202 | .841 |
|  | (Constant) | .456 | 1.477 |  | .309 | .758 |
|  | **FFS** | **.065** | **.028** | **.256** | **2.309** | **.023** |
|  | Age | -.068 | .056 | -.139 | -1.223 | .225 |
|  | Sex | -.158 | .223 | -.079 | -.709 | .480 |
|  | Subjective SES | .005 | .067 | .008 | .074 | .941 |
| Life satisfaction | (Constant) | 6.751 | 1.867 |  | 3.616 | .001 |
|  | **MA** | **.063** | **.022** | **.349** | **2.917** | **.005** |
|  | Age | -.077 | .073 | -.119 | -1.055 | .294 |
|  | Sex | -.223 | .287 | -.085 | -.777 | .439 |
|  | Subjective SES | -.037 | .093 | -.047 | -.397 | .693 |
|  | (Constant) | 8.679 | 1.835 |  | 4.730 | .000 |
|  | **CMS** | **-.072** | **.032** | **-.257** | **-2.206** | **.030** |
|  | Age | -.075 | .075 | -.116 | -.995 | .322 |
|  | Sex | -.190 | .293 | -.072 | -.647 | .520 |
|  | Subjective SES | .020 | .089 | .026 | .228 | .820 |
|  | (Constant) | 7.034 | 1.958 |  | 3.592 | .001 |
|  | **FFS** | **.071** | **.038** | **.212** | **1.899** | **.061** |
|  | Age | -.098 | .074 | -.151 | -1.316 | .192 |
|  | Sex | -.184 | .295 | -.070 | -.621 | .536 |
|  | Subjective SES | .037 | .089 | .047 | .418 | .677 |
| Feeling of happiness | (Constant) | 6.630 | 1.856 |  | 3.572 | .001 |
|  | **MA** | **.054** | **.022** | **.308** | **2.523** | **.014** |
|  | Age | -.067 | .072 | -.107 | -.930 | .355 |
|  | Sex | -.208 | .286 | -.081 | -.729 | .468 |
|  | Subjective SES | -.067 | .092 | -.088 | -.728 | .469 |
|  | (Constant) | 8.348 | 1.798 |  | 4.644 | .000 |
|  | **CMS** | **-.073** | **.032** | **-.270** | **-2.302** | **.024** |
|  | Age | -.059 | .074 | -.094 | -.801 | .426 |
|  | Sex | -.177 | .287 | -.069 | -.616 | .540 |
|  | Subjective SES | -.028 | .088 | -.037 | -.322 | .748 |
|  | (Constant) | 6.390 | 1.898 |  | 3.367 | .001 |
|  | **FFS** | **.088** | **.036** | **.270** | **2.431** | **.017** |
|  | Age | -.078 | .072 | -.124 | -1.090 | .279 |
|  | Sex | -.167 | .286 | -.065 | -.583 | .562 |
|  | Subjective SES | -.021 | .086 | -.028 | -.246 | .807 |
| Meaning in life | (Constant) | 6.655 | 1.879 |  | 3.541 | .001 |
|  | **MA** | **.068** | **.022** | **.368** | **3.116** | **.003** |
|  | Age | -.092 | .073 | -.139 | -1.253 | .214 |
|  | Sex | -.065 | .289 | -.024 | -.225 | .823 |
|  | Subjective SES | -.047 | .093 | -.059 | -.509 | .612 |
|  | (Constant) | 8.775 | 1.836 |  | 4.779 | .000 |
|  | **CMS** | **-.086** | **.032** | **-.303** | **-2.653** | **.010** |
|  | Age | -.085 | .075 | -.128 | -1.122 | .265 |
|  | Sex | -.027 | .293 | -.010 | -.093 | .926 |
|  | Subjective SES | .006 | .089 | .007 | .066 | .947 |
|  | (Constant) | 7.092 | 1.987 |  | 3.569 | .001 |
|  | **FFS** | **.069** | **.038** | **.202** | **1.816** | **.073** |
|  | Age | -.116 | .075 | -.176 | -1.541 | .127 |
|  | Sex | -.024 | .300 | -.009 | -.081 | .936 |
|  | Subjective SES | .037 | .090 | .046 | .409 | .684 |
| Stress | (Constant) | 5.636 | 1.922 |  | 2.933 | .004 |
|  | **MA** | **-.081** | **.022** | **-.430** | **-3.652** | **.000** |
|  | Age | .009 | .075 | .014 | .127 | .899 |
|  | Sex | .483 | .296 | .176 | 1.635 | .106 |
|  | Subjective SES | .128 | .095 | .156 | 1.340 | .184 |
|  | (Constant) | 3.043 | 1.865 |  | 1.631 | .107 |
|  | **CMS** | **.113** | **.033** | **.388** | **3.433** | **.001** |
|  | Age | -.005 | .077 | -.007 | -.063 | .950 |
|  | Sex | .436 | .298 | .159 | 1.464 | .147 |
|  | Subjective SES | .073 | .091 | .089 | .799 | .426 |
|  | (Constant) | 5.279 | 2.045 |  | 2.581 | .012 |
|  | **FFS** | **-.092** | **.039** | **-.263** | **-2.354** | **.021** |
|  | Age | .036 | .077 | .053 | .468 | .641 |
|  | Sex | .432 | .309 | .157 | 1.400 | .165 |
|  | Subjective SES | .033 | .093 | .040 | .355 | .724 |

*Note*. CMS, current money management stress; FFS, future financial security; MA, material affluence; SES, socioeconomic status.

**Supplementary Table 4*.** Regression results of FWB as the predictor, momentary happiness as the outcome variable, and age, sex, and subjective SES as covariates (*N* = 54).

| **Dependent**  **Variables** | **Independent**  **Variables** | **B** | ***SE*** | ***β*** | ***t*** | ***p*** |
| --- | --- | --- | --- | --- | --- | --- |
| Composite score  of happiness | (Constant) | 1.441 | 1.700 |  | .847 | .401 |
|  | **MA** | **.061** | **.022** | **.404** | **2.757** | **.008** |
|  | Age | -.096 | .062 | -.216 | -1.534 | .131 |
|  | Sex | -.283 | .287 | -.131 | -.984 | .330 |
|  | Subjective SES | -.082 | .085 | -.137 | -.964 | .340 |
|  | (Constant) | 3.576 | 1.573 |  | 2.273 | .027 |
|  | **CMS** | **-.087** | **.030** | **-.421** | **-2.930** | **.005** |
|  | Age | -.084 | .063 | -.189 | -1.333 | .189 |
|  | Sex | -.198 | .285 | -.092 | -.696 | .490 |
|  | Subjective SES | -.075 | .083 | -.125 | -.902 | .371 |
|  | (Constant) | 1.624 | 1.713 |  | .948 | .348 |
|  | **FFS** | **.085** | **.034** | **.347** | **2.511** | **.015** |
|  | Age | -.112 | .062 | -.254 | -1.812 | .076 |
|  | Sex | -.221 | .290 | -.102 | -.761 | .450 |
|  | Subjective SES | -.052 | .083 | -.087 | -.629 | .532 |
| Life satisfaction | (Constant) | 8.162 | 2.295 |  | 3.556 | .001 |
|  | **MA** | **.070** | **.030** | **.353** | **2.350** | **.023** |
|  | Age | -.122 | .084 | -.209 | -1.450 | .154 |
|  | Sex | -.431 | .388 | -.151 | -1.113 | .271 |
|  | Subjective SES | -.095 | .115 | -.120 | -.826 | .413 |
|  | (Constant) | 10.605 | 2.136 |  | 4.964 | .000 |
|  | **CMS** | **-.098** | **.040** | **-.358** | **-2.414** | **.020** |
|  | Age | -.110 | .085 | -.188 | -1.290 | .203 |
|  | Sex | -.336 | .387 | -.118 | -.868 | .390 |
|  | Subjective SES | -.084 | .113 | -.106 | -.745 | .460 |
|  | (Constant) | 8.206 | 2.284 |  | 3.593 | .001 |
|  | **FFS** | **.107** | **.045** | **.331** | **2.371** | **.022** |
|  | Age | -.138 | .083 | -.237 | -1.675 | .100 |
|  | Sex | -.358 | .387 | -.126 | -.925 | .359 |
|  | Subjective SES | -.068 | .111 | -.085 | -.609 | .545 |
| Feeling of happiness | (Constant) | 8.048 | 2.289 |  | 3.516 | .001 |
|  | **MA** | **.061** | **.030** | **.311** | **2.036** | **.047** |
|  | Age | -.122 | .084 | -.214 | -1.461 | .151 |
|  | Sex | -.290 | .387 | -.104 | -.750 | .457 |
|  | Subjective SES | -.088 | .115 | -.113 | -.762 | .450 |
|  | (Constant) | 10.231 | 2.093 |  | 4.888 | .000 |
|  | **CMS** | **-.100** | **.040** | **-.373** | **-2.520** | **.015** |
|  | Age | -.102 | .083 | -.179 | -1.228 | .225 |
|  | Sex | -.199 | .379 | -.071 | -.526 | .601 |
|  | Subjective SES | -.093 | .111 | -.120 | -.843 | .403 |
|  | (Constant) | 7.715 | 2.229 |  | 3.461 | .001 |
|  | **FFS** | **.113** | **.044** | **.356** | **2.562** | **.014** |
|  | Age | -.131 | .081 | -.228 | -1.620 | .112 |
|  | Sex | -.221 | .378 | -.079 | -.586 | .560 |
|  | Subjective SES | -.079 | .108 | -.101 | -.728 | .470 |
| Meaning in life | (Constant) | 7.685 | 2.383 |  | 3.225 | .002 |
|  | **MA** | **.084** | **.031** | **.397** | **2.696** | **.010** |
|  | Age | -.129 | .087 | -.209 | -1.482 | .145 |
|  | Sex | -.162 | .402 | -.054 | -.402 | .689 |
|  | Subjective SES | -.112 | .120 | -.133 | -.934 | .355 |
|  | (Constant) | 10.592 | 2.219 |  | 4.773 | .000 |
|  | **CMS** | **-.115** | **.042** | **-.399** | **-2.745** | **.008** |
|  | Age | -.116 | .088 | -.187 | -1.307 | .197 |
|  | Sex | -.048 | .402 | -.016 | -.120 | .905 |
|  | Subjective SES | -.098 | .117 | -.116 | -.832 | .410 |
|  | (Constant) | 8.509 | 2.468 |  | 3.447 | .001 |
|  | **FFS** | **.085** | **.049** | **.248** | **1.742** | **.088** |
|  | Age | -.162 | .089 | -.262 | -1.811 | .076 |
|  | Sex | -.085 | .418 | -.028 | -.203 | .840 |
|  | Subjective SES | -.047 | .120 | -.056 | -.395 | .695 |
| Stress | (Constant) | 3.856 | 2.136 |  | 1.805 | .077 |
|  | **MA** | **-.087** | **.028** | **-.456** | **-3.147** | **.003** |
|  | Age | .089 | .078 | .159 | 1.142 | .259 |
|  | Sex | .521 | .361 | .190 | 1.445 | .155 |
|  | Subjective SES | .111 | .107 | .146 | 1.037 | .305 |
|  | (Constant) | .833 | 2.000 |  | .416 | .679 |
|  | CMS | **.117** | **.038** | **.444** | **3.087** | **.003** |
|  | Age | .077 | .080 | .137 | .969 | .337 |
|  | Sex | .404 | .362 | .147 | 1.118 | .269 |
|  | Subjective SES | .093 | .106 | .121 | .877 | .385 |
|  | (Constant) | 3.388 | 2.191 |  | 1.546 | .129 |
|  | **FFS** | **-.111** | **.043** | **-.355** | **-2.554** | **.014** |
|  | Age | .117 | .079 | .207 | 1.472 | .147 |
|  | Sex | .436 | .371 | .159 | 1.174 | .246 |
|  | Subjective SES | .060 | .107 | .078 | .563 | .576 |

*Note*. CMS, current money management stress; FFS, future financial security; MA, material affluence; SES, socioeconomic status.

**Supplementary Table 5-1.** Regression results of the anticipatory responses when expecting upcoming positive events as the predictor, momentary happiness as the outcome variable, and age, sex, subjective SES, and intercept of each affective state (if applicable) as covariates.

| **Dependent**  **Variables** | **Independent**  **Variables** | **B** | ***SE*** | ***β*** | ***t*** | ***p*** |
| --- | --- | --- | --- | --- | --- | --- |
| Composite score  of happiness | (Constant) | -4.238 | .583 |  | -7.270 | .000 |
|  | **Valence** | **.413** | **.127** | **.134** | **3.249** | **.002** |
|  | Age | -.068 | .021 | -.139 | -3.234 | .002 |
|  | Sex | -.112 | .086 | -.056 | -1.299 | .198 |
|  | Subjective SES | -.017 | .025 | -.029 | -.703 | .484 |
|  | Intercept | 1.016 | .046 | .894 | 22.163 | .000 |
|  | (Constant) | -3.574 | .927 |  | -3.854 | .000 |
|  | **Interestedness** | **.937** | **.357** | **.200** | **2.623** | **.010** |
|  | Age | -.086 | .033 | -.174 | -2.587 | .011 |
|  | Sex | -.174 | .134 | -.087 | -1.302 | .197 |
|  | Subjective SES | -.025 | .039 | -.042 | -.640 | .524 |
|  | Intercept | .984 | .111 | .682 | 8.902 | .000 |
|  | (Constant) | -3.274 | 1.118 |  | -2.929 | .004 |
|  | **Activeness** | **.909** | **.562** | **.193** | **1.617** | **.110** |
|  | Age | -.066 | .039 | -.135 | -1.710 | .091 |
|  | Sex | -.180 | .155 | -.090 | -1.157 | .251 |
|  | Subjective SES | .012 | .045 | .020 | .258 | .797 |
|  | Intercept | .762 | .160 | .570 | 4.765 | .000 |
| Life satisfaction | (Constant) | .809 | .859 |  | .941 | .349 |
|  | **Valence** | **.674** | **.187** | **.166** | **3.598** | **.001** |
|  | Age | -.097 | .031 | -.150 | -3.127 | .002 |
|  | Sex | -.141 | .127 | -.054 | -1.112 | .269 |
|  | Subjective SES | -.002 | .036 | -.003 | -.059 | .953 |
|  | Intercept | 1.291 | .068 | .862 | 19.111 | .000 |
|  | (Constant) | 1.862 | 1.347 |  | 1.382 | .171 |
|  | **Interestedness** | **1.123** | **.519** | **.182** | **2.165** | **.033** |
|  | Age | -.116 | .048 | -.180 | -2.423 | .018 |
|  | Sex | -.200 | .195 | -.076 | -1.026 | .308 |
|  | Subjective SES | -.006 | .056 | -.008 | -.110 | .913 |
|  | Intercept | 1.227 | .161 | .645 | 7.640 | .000 |
|  | (Constant) | 2.212 | 1.523 |  | 1.452 | .150 |
|  | **Activeness** | **1.401** | **.766** | **.225** | **1.829** | **.071** |
|  | Age | -.093 | .053 | -.143 | -1.754 | .083 |
|  | Sex | -.210 | .212 | -.080 | -.992 | .324 |
|  | Subjective SES | .038 | .061 | .049 | .625 | .533 |
|  | Intercept | .905 | .218 | .513 | 4.150 | .000 |
| Feeling of happiness | (Constant) | .406 | .741 |  | .547 | .586 |
|  | **Valence** | **.573** | **.162** | **.145** | **3.545** | **.001** |
|  | Age | -.080 | .027 | -.127 | -2.982 | .004 |
|  | Sex | -.114 | .109 | -.044 | -1.039 | .302 |
|  | Subjective SES | -.048 | .031 | -.063 | -1.535 | .129 |
|  | Intercept | 1.309 | .058 | .898 | 22.453 | .000 |
|  | (Constant) | 1.483 | 1.248 |  | 1.189 | .238 |
|  | **Interestedness** | **1.386** | **.481** | **.231** | **2.882** | **.005** |
|  | Age | -.102 | .045 | -.161 | -2.287 | .025 |
|  | Sex | -.200 | .180 | -.078 | -1.107 | .272 |
|  | Subjective SES | -.054 | .052 | -.070 | -1.029 | .307 |
|  | Intercept | 1.193 | .149 | .645 | 8.021 | .000 |
|  | (Constant) | 1.948 | 1.486 |  | 1.311 | .193 |
|  | **Activeness** | **1.398** | **.747** | **.231** | **1.871** | **.065** |
|  | Age | -.078 | .051 | -.124 | -1.514 | .134 |
|  | Sex | -.198 | .206 | -.077 | -.958 | .341 |
|  | Subjective SES | -.008 | .060 | -.011 | -.138 | .891 |
|  | Intercept | .891 | .213 | .519 | 4.191 | .000 |
| Meaning in life | (Constant) | 1.631 | 1.282 |  | 1.273 | .207 |
|  | **Valence** | **.436** | **.279** | **.105** | **1.562** | **.122** |
|  | Age | -.114 | .046 | -.172 | -2.451 | .016 |
|  | Sex | .035 | .189 | .013 | .185 | .854 |
|  | Subjective SES | .009 | .054 | .011 | .159 | .874 |
|  | Intercept | 1.163 | .101 | .761 | 11.538 | .000 |
|  | (Constant) | 1.986 | 1.410 |  | 1.409 | .163 |
|  | **Interestedness** | **1.228** | **.543** | **.195** | **2.261** | **.026** |
|  | Age | -.134 | .050 | -.203 | -2.672 | .009 |
|  | Sex | -.046 | .204 | -.017 | -.224 | .823 |
|  | Subjective SES | -.006 | .059 | -.008 | -.107 | .915 |
|  | Intercept | 1.187 | .168 | .612 | 7.061 | .000 |
|  | (Constant) | 1.482 | 1.415 |  | 1.048 | .298 |
|  | **Activeness** | **1.067** | **.711** | **.168** | **1.500** | **.137** |
|  | Age | -.107 | .049 | -.162 | -2.187 | .032 |
|  | Sex | -.047 | .196 | -.018 | -.240 | .811 |
|  | Subjective SES | .032 | .057 | .040 | .564 | .574 |
|  | Intercept | 1.099 | .202 | .611 | 5.427 | .000 |
| Stress | (Constant) | 10.438 | 1.402 |  | 7.446 | .000 |
|  | **Valence** | **-.290** | **.306** | **-.068** | **-.948** | **.346** |
|  | Age | .036 | .051 | .054 | .716 | .476 |
|  | Sex | .356 | .207 | .130 | 1.725 | .088 |
|  | Subjective SES | .044 | .059 | .054 | .752 | .454 |
|  | Intercept | -1.191 | .110 | -.760 | -10.802 | .000 |
|  | (Constant) | 9.735 | 1.633 |  | 5.961 | .000 |
|  | **Interestedness** | **-.777** | **.629** | **-.120** | **-1.234** | **.221** |
|  | Age | .060 | .058 | .088 | 1.028 | .307 |
|  | Sex | .440 | .236 | .160 | 1.863 | .066 |
|  | Subjective SES | .058 | .068 | .071 | .848 | .399 |
|  | Intercept | -1.208 | .195 | -.608 | -6.203 | .000 |
|  | (Constant) | 8.476 | 1.988 |  | 4.263 | .000 |
|  | **Activeness** | **-.444** | **1.000** | **-.068** | **-.444** | **.658** |
|  | Age | .041 | .069 | .060 | .591 | .556 |
|  | Sex | .457 | .276 | .166 | 1.654 | .102 |
|  | Subjective SES | .008 | .080 | .009 | .095 | .925 |
|  | Intercept | -.828 | .285 | -.449 | -2.911 | .005 |

*Note*. SES, socioeconomic status.

**Supplementary Table 5-2.** Regression results of the anticipatory responses when expecting upcoming negative events as the predictor, momentary happiness as the outcome variables, and age, sex, subjective SES, and intercept of each affective state (if applicable) as covariates.

| **Dependent**  **Variables** | **Independent**  **Variables** | **B** | ***SE*** | ***β*** | ***t*** | ***p*** |
| --- | --- | --- | --- | --- | --- | --- |
| Composite score  of happiness | (Constant) | -4.446 | .767 |  | -5.798 | .000 |
|  | **Valence** | **-.348** | **.104** | **-.279** | **-3.357** | **.001** |
|  | Age | -.055 | .026 | -.112 | -2.139 | .035 |
|  | Sex | -.060 | .106 | -.030 | -.559 | .578 |
|  | Subjective SES | -.009 | .030 | -.015 | -.307 | .760 |
|  | Intercept | 1.082 | .079 | 1.093 | 13.648 | .000 |
|  | (Constant) | -4.449 | 1.152 |  | -3.862 | .000 |
|  | **Interestedness** | **-.432** | **.153** | **-.361** | **-2.825** | **.006** |
|  | Age | -.076 | .036 | -.154 | -2.123 | .037 |
|  | Sex | -.124 | .146 | -.062 | -.850 | .398 |
|  | Subjective SES | -.019 | .042 | -.032 | -.460 | .647 |
|  | Intercept | 1.270 | .150 | 1.062 | 8.453 | .000 |
|  | (Constant) | -3.404 | 1.114 |  | -3.057 | .003 |
|  | **Activeness** | **-.342** | **.144** | **-.222** | **-2.367** | **.020** |
|  | Age | -.059 | .039 | -.120 | -1.498 | .138 |
|  | Sex | -.153 | .159 | -.076 | -.958 | .341 |
|  | Subjective SES | -.005 | .046 | -.009 | -.111 | .912 |
|  | Intercept | .945 | .106 | .836 | 8.909 | .000 |
| Life satisfaction | (Constant) | .680 | 1.063 |  | .639 | .525 |
|  | **Valence** | **-.372** | **.144** | **-.226** | **-2.583** | **.012** |
|  | Age | -.081 | .036 | -.125 | -2.265 | .026 |
|  | Sex | -.078 | .148 | -.030 | -.529 | .598 |
|  | Subjective SES | .005 | .042 | .007 | .129 | .898 |
|  | Intercept | 1.358 | .110 | 1.041 | 12.356 | .000 |
|  | (Constant) | .297 | 1.605 |  | .185 | .853 |
|  | **Interestedness** | **-.647** | **.213** | **-.410** | **-3.033** | **.003** |
|  | Age | -.101 | .050 | -.156 | -2.031 | .045 |
|  | Sex | -.114 | .204 | -.043 | -.560 | .577 |
|  | Subjective SES | .003 | .058 | .004 | .052 | .959 |
|  | Intercept | 1.670 | .209 | 1.060 | 7.980 | .000 |
|  | (Constant) | 1.841 | 1.498 |  | 1.229 | .222 |
|  | **Activeness** | **-.475** | **.194** | **-.234** | **-2.443** | **.017** |
|  | Age | -.082 | .053 | -.127 | -1.550 | .125 |
|  | Sex | -.168 | .214 | -.064 | -.784 | .435 |
|  | Subjective SES | .016 | .062 | .020 | .254 | .800 |
|  | Intercept | 1.223 | .143 | .821 | 8.573 | .000 |
| Feeling of happiness | (Constant) | .293 | .953 |  | .308 | .759 |
|  | **Valence** | **-.378** | **.129** | **-.236** | **-2.934** | **.004** |
|  | Age | -.065 | .032 | -.104 | -2.046 | .044 |
|  | Sex | -.064 | .132 | -.025 | -.481 | .632 |
|  | Subjective SES | -.042 | .037 | -.055 | -1.119 | .266 |
|  | Intercept | 1.367 | .099 | 1.076 | 13.869 | .000 |
|  | (Constant) | .595 | 1.504 |  | .395 | .694 |
|  | **Interestedness** | **-.443** | **.200** | **-.289** | **-2.217** | **.029** |
|  | Age | -.092 | .047 | -.146 | -1.980 | .051 |
|  | Sex | -.151 | .191 | -.059 | -.789 | .432 |
|  | Subjective SES | -.053 | .054 | -.069 | -.965 | .338 |
|  | Intercept | 1.546 | .196 | 1.009 | 7.883 | .000 |
|  | (Constant) | 1.679 | 1.468 |  | 1.143 | .256 |
|  | **Activeness** | **-.315** | **.191** | **-.159** | **-1.653** | **.102** |
|  | Age | -.069 | .052 | -.109 | -1.326 | .189 |
|  | Sex | -.179 | .210 | -.070 | -.854 | .396 |
|  | Subjective SES | -.029 | .061 | -.038 | -.474 | .637 |
|  | Intercept | 1.157 | .140 | .798 | 8.272 | .000 |
| Meaning in life | (Constant) | 1.912 | 1.467 |  | 1.304 | .196 |
|  | **Valence** | **-.210** | **.198** | **-.125** | **-1.058** | **.293** |
|  | Age | -.108 | .049 | -.164 | -2.204 | .030 |
|  | Sex | .029 | .204 | .011 | .144 | .886 |
|  | Subjective SES | .005 | .057 | .007 | .094 | .925 |
|  | Intercept | 1.140 | .152 | .857 | 7.519 | .000 |
|  | (Constant) | .876 | 1.715 |  | .511 | .611 |
|  | **Interestedness** | **-.535** | **.228** | **-.332** | **-2.346** | **.021** |
|  | Age | -.122 | .053 | -.185 | -2.299 | .024 |
|  | Sex | .021 | .218 | .008 | .098 | .922 |
|  | Subjective SES | .000 | .062 | .001 | .008 | .994 |
|  | Intercept | 1.558 | .224 | .970 | 6.965 | .000 |
|  | (Constant) | 1.349 | 1.403 |  | .962 | .339 |
|  | **Activeness** | **-.480** | **.182** | **-.232** | **-2.638** | **.010** |
|  | Age | -.098 | .050 | -.148 | -1.965 | .053 |
|  | Sex | -.010 | .200 | -.004 | -.051 | .959 |
|  | Subjective SES | .009 | .058 | .011 | .154 | .878 |
|  | Intercept | 1.313 | .134 | .865 | 9.827 | .000 |
| Stress | (Constant) | 11.639 | 1.623 |  | 7.173 | .000 |
|  | **Valence** | **.800** | **.220** | **.465** | **3.645** | **.000** |
|  | Age | .006 | .054 | .009 | .113 | .911 |
|  | Sex | .195 | .225 | .071 | .865 | .390 |
|  | Subjective SES | .012 | .064 | .015 | .190 | .850 |
|  | Intercept | -1.435 | .168 | -1.051 | -8.553 | .000 |
|  | (Constant) | 10.383 | 2.020 |  | 5.140 | .000 |
|  | **Interestedness** | **.485** | **.268** | **.294** | **1.808** | **.074** |
|  | Age | .049 | .062 | .072 | .785 | .435 |
|  | Sex | .398 | .256 | .145 | 1.554 | .124 |
|  | Subjective SES | .047 | .073 | .057 | .637 | .526 |
|  | Intercept | -1.411 | .263 | -.857 | -5.358 | .000 |
|  | (Constant) | 8.266 | 1.981 |  | 4.172 | .000 |
|  | **Activeness** | **.409** | **.257** | **.193** | **1.593** | **.115** |
|  | Age | .035 | .070 | .052 | .502 | .617 |
|  | Sex | .421 | .283 | .153 | 1.485 | .141 |
|  | Subjective SES | .022 | .082 | .027 | .269 | .789 |
|  | Intercept | -.892 | .189 | -.573 | -4.726 | .000 |

*Note*. SES, socioeconomic status.

**Supplementary Table 5-1*.** Regression results of the anticipatory responses when expecting upcoming positive events as the predictor, momentary happiness as the outcome variable, and age, sex, subjective SES, and intercept of each affective state (if applicable) as covariates (*N* =54).

| **Dependent**  **Variables** | **Independent**  **Variables** | **B** | ***SE*** | ***β*** | ***t*** | ***p*** |
| --- | --- | --- | --- | --- | --- | --- |
| Composite score  of happiness | (Constant) | -3.942 | .773 |  | -5.103 | .000 |
|  | **Valence** | **.505** | **.162** | **.172** | **3.127** | **.003** |
|  | Age | -.081 | .025 | -.183 | -3.228 | .002 |
|  | Sex | -.104 | .118 | -.048 | -.877 | .385 |
|  | Subjective SES | -.037 | .033 | -.061 | -1.135 | .262 |
|  | Intercept | 1.022 | .064 | .851 | 16.028 | .000 |
|  | (Constant) | -2.421 | 1.208 |  | -2.005 | .051 |
|  | **Interestedness** | **1.570** | **.477** | **.329** | **3.293** | **.002** |
|  | Age | -.108 | .039 | -.244 | -2.797 | .007 |
|  | Sex | -.294 | .183 | -.136 | -1.603 | .115 |
|  | Subjective SES | -.088 | .051 | -.146 | -1.708 | .094 |
|  | Intercept | .833 | .146 | .564 | 5.717 | .000 |
|  | (Constant) | -2.416 | 1.341 |  | -1.801 | .078 |
|  | **Activeness** | **1.006** | **.679** | **.221** | **1.482** | **.145** |
|  | Age | -.091 | .041 | -.206 | -2.235 | .030 |
|  | Sex | -.261 | .188 | -.121 | -1.387 | .172 |
|  | Subjective SES | -.035 | .053 | -.058 | -.655 | .515 |
|  | Intercept | .756 | .193 | .584 | 3.926 | .000 |
| Life satisfaction | (Constant) | 1.158 | 1.056 |  | 1.096 | .278 |
|  | **Valence** | **.961** | **.221** | **.248** | **4.348** | **.000** |
|  | Age | -.107 | .034 | -.183 | -3.122 | .003 |
|  | Sex | -.258 | .162 | -.091 | -1.597 | .117 |
|  | Subjective SES | -.066 | .044 | -.083 | -1.489 | .143 |
|  | Intercept | 1.294 | .087 | .818 | 14.854 | .000 |
|  | (Constant) | 3.299 | 1.763 |  | 1.872 | .067 |
|  | **Interestedness** | **2.133** | **.696** | **.339** | **3.064** | **.004** |
|  | Age | -.135 | .056 | -.231 | -2.390 | .021 |
|  | Sex | -.458 | .268 | -.161 | -1.711 | .094 |
|  | Subjective SES | -.113 | .075 | -.143 | -1.513 | .137 |
|  | Intercept | .995 | .213 | .511 | 4.679 | .000 |
|  | (Constant) | 3.769 | 1.893 |  | 1.991 | .052 |
|  | **Activeness** | **2.050** | **.958** | **.342** | **2.140** | **.037** |
|  | Age | -.121 | .058 | -.208 | -2.109 | .040 |
|  | Sex | -.427 | .266 | -.150 | -1.608 | .114 |
|  | Subjective SES | -.061 | .075 | -.076 | -.807 | .424 |
|  | Intercept | .756 | .272 | .443 | 2.782 | .008 |
| Feeling of happiness | (Constant) | .525 | 1.008 |  | .521 | .605 |
|  | **Valence** | **.690** | **.211** | **.182** | **3.272** | **.002** |
|  | Age | -.091 | .033 | -.160 | -2.798 | .007 |
|  | Sex | -.075 | .154 | -.027 | -.485 | .630 |
|  | Subjective SES | -.059 | .042 | -.076 | -1.387 | .172 |
|  | Intercept | 1.328 | .083 | .855 | 15.974 | .000 |
|  | (Constant) | 2.889 | 1.641 |  | 1.760 | .085 |
|  | **Interestedness** | **2.318** | **.648** | **.376** | **3.577** | **.001** |
|  | Age | -.129 | .053 | -.226 | -2.462 | .017 |
|  | Sex | -.331 | .249 | -.119 | -1.329 | .190 |
|  | Subjective SES | -.125 | .070 | -.160 | -1.789 | .080 |
|  | Intercept | .976 | .198 | .511 | 4.933 | .000 |
|  | (Constant) | 2.874 | 1.787 |  | 1.608 | .114 |
|  | **Activeness** | **1.626** | **.904** | **.276** | **1.797** | **.079** |
|  | Age | -.109 | .054 | -.190 | -2.001 | .051 |
|  | Sex | -.282 | .251 | -.101 | -1.124 | .267 |
|  | Subjective SES | -.060 | .071 | -.077 | -.847 | .401 |
|  | Intercept | .887 | .257 | .529 | 3.457 | .001 |
| Meaning in life | (Constant) | 1.064 | 1.627 |  | .654 | .516 |
|  | **Valence** | **.558** | **.340** | **.136** | **1.640** | **.108** |
|  | Age | -.113 | .053 | -.183 | -2.147 | .037 |
|  | Sex | .081 | .249 | .027 | .324 | .747 |
|  | Subjective SES | -.040 | .068 | -.048 | -.584 | .562 |
|  | Intercept | 1.291 | .134 | .770 | 9.620 | .000 |
|  | (Constant) | 2.667 | 1.798 |  | 1.483 | .145 |
|  | **Interestedness** | **2.307** | **.710** | **.346** | **3.250** | **.002** |
|  | Age | -.149 | .058 | -.241 | -2.587 | .013 |
|  | Sex | -.186 | .273 | -.062 | -.680 | .500 |
|  | Subjective SES | -.119 | .076 | -.142 | -1.563 | .125 |
|  | Intercept | 1.071 | .217 | .519 | 4.941 | .000 |
|  | (Constant) | 1.942 | 1.764 |  | 1.101 | .276 |
|  | **Activeness** | **1.388** | **.893** | **.219** | **1.555** | **.126** |
|  | Age | -.119 | .054 | -.193 | -2.222 | .031 |
|  | Sex | -.132 | .248 | -.044 | -.534 | .596 |
|  | Subjective SES | -.049 | .070 | -.058 | -.697 | .489 |
|  | Intercept | 1.107 | .253 | .612 | 4.370 | .000 |
| Stress | (Constant) | 8.693 | 1.892 |  | 4.595 | .000 |
|  | **Valence** | **-.175** | **.396** | **-.047** | **-.442** | **.661** |
|  | Age | .082 | .061 | .146 | 1.342 | .186 |
|  | Sex | .269 | .290 | .098 | .928 | .358 |
|  | Subjective SES | .008 | .080 | .010 | .097 | .923 |
|  | Intercept | -1.049 | .156 | -.687 | -6.717 | .000 |
|  | (Constant) | 7.522 | 2.042 |  | 3.684 | .001 |
|  | **Interestedness** | **-.712** | **.806** | **-.117** | **-.884** | **.381** |
|  | Age | .114 | .065 | .202 | 1.739 | .088 |
|  | Sex | .474 | .310 | .172 | 1.528 | .133 |
|  | Subjective SES | .063 | .087 | .083 | .730 | .469 |
|  | Intercept | -1.040 | .246 | -.554 | -4.224 | .000 |
|  | (Constant) | 7.222 | 2.405 |  | 3.003 | .004 |
|  | **Activeness** | **.462** | **1.217** | **.080** | **.379** | **.706** |
|  | Age | .094 | .073 | .167 | 1.284 | .205 |
|  | Sex | .448 | .338 | .163 | 1.327 | .191 |
|  | Subjective SES | -.009 | .095 | -.011 | -.090 | .929 |
|  | Intercept | -.972 | .345 | -.590 | -2.815 | .007 |

*Note*. SES, socioeconomic status.

**Supplementary Table 5-2*.** Regression results of the anticipatory responses when expecting upcoming negative events as the predictor, momentary happiness as the outcome variable, and age, sex, subjective SES, and intercept of each affective state (if applicable) as covariates (*N* = 54).

| **Dependent**  **Variables** | **Independent**  **Variables** | **B** | ***SE*** | ***β*** | ***t*** | ***p*** |
| --- | --- | --- | --- | --- | --- | --- |
| Composite score  of happiness | (Constant) | -4.399 | 1.065 |  | -4.132 | .000 |
|  | **Valence** | **-.445** | **.130** | **-.376** | **-3.410** | **.001** |
|  | Age | -.062 | .032 | -.141 | -1.945 | .058 |
|  | Sex | -.174 | .151 | -.080 | -1.150 | .256 |
|  | Subjective SES | -.048 | .041 | -.081 | -1.184 | .242 |
|  | Intercept | 1.173 | .114 | 1.131 | 10.320 | .000 |
|  | (Constant) | -4.299 | 1.539 |  | -2.793 | .007 |
|  | **Interestedness** | **-.611** | **.187** | **-.525** | **-3.263** | **.002** |
|  | Age | -.087 | .043 | -.196 | -2.004 | .051 |
|  | Sex | -.282 | .206 | -.130 | -1.368 | .178 |
|  | Subjective SES | -.062 | .057 | -.102 | -1.073 | .289 |
|  | Intercept | 1.379 | .196 | 1.120 | 7.049 | .000 |
|  | (Constant) | -3.338 | 1.287 |  | -2.593 | .013 |
|  | **Activeness** | **-.557** | **.174** | **-.358** | **-3.192** | **.002** |
|  | Age | -.066 | .041 | -.148 | -1.592 | .118 |
|  | Sex | -.279 | .193 | -.129 | -1.442 | .156 |
|  | Subjective SES | -.032 | .053 | -.052 | -.591 | .557 |
|  | Intercept | 1.048 | .123 | .957 | 8.507 | .000 |
| Life satisfaction | (Constant) | .245 | 1.317 |  | .186 | .853 |
|  | **Valence** | **-.501** | **.161** | **-.321** | **-3.104** | **.003** |
|  | Age | -.074 | .040 | -.127 | -1.862 | .069 |
|  | Sex | -.320 | .187 | -.112 | -1.714 | .093 |
|  | Subjective SES | -.073 | .051 | -.092 | -1.443 | .156 |
|  | Intercept | 1.527 | .141 | 1.117 | 10.861 | .000 |
|  | (Constant) | .323 | 2.105 |  | .153 | .879 |
|  | **Interestedness** | **-.844** | **.256** | **-.550** | **-3.294** | **.002** |
|  | Age | -.103 | .059 | -.176 | -1.734 | .089 |
|  | Sex | -.422 | .282 | -.148 | -1.498 | .141 |
|  | Subjective SES | -.077 | .078 | -.098 | -.986 | .329 |
|  | Intercept | 1.814 | .268 | 1.118 | 6.778 | .000 |
|  | (Constant) | 1.874 | 1.792 |  | 1.045 | .301 |
|  | **Activeness** | **-.645** | **.243** | **-.315** | **-2.657** | **.011** |
|  | Age | -.080 | .057 | -.137 | -1.389 | .171 |
|  | Sex | -.445 | .269 | -.156 | -1.653 | .105 |
|  | Subjective SES | -.047 | .074 | -.059 | -.627 | .534 |
|  | Intercept | 1.327 | .171 | .920 | 7.739 | .000 |
| Feeling of happiness | (Constant) | .189 | 1.347 |  | .140 | .889 |
|  | **Valence** | **-.463** | **.165** | **-.302** | **-2.803** | **.007** |
|  | Age | -.072 | .041 | -.125 | -1.765 | .084 |
|  | Sex | -.194 | .191 | -.069 | -1.014 | .316 |
|  | Subjective SES | -.079 | .052 | -.102 | -1.529 | .133 |
|  | Intercept | 1.474 | .144 | 1.099 | 10.249 | .000 |
|  | (Constant) | .189 | 1.347 |  | .140 | .889 |
|  | **Interestedness** | **-.463** | **.165** | **-.302** | **-2.803** | **.007** |
|  | Age | -.072 | .041 | -.125 | -1.765 | .084 |
|  | Sex | -.194 | .191 | -.069 | -1.014 | .316 |
|  | Subjective SES | -.079 | .052 | -.102 | -1.529 | .133 |
|  | Intercept | 1.474 | .144 | 1.099 | 10.249 | .000 |
|  | (Constant) | 1.695 | 1.732 |  | .979 | .333 |
|  | **Activeness** | **-.497** | **.235** | **-.247** | **-2.117** | **.039** |
|  | Age | -.078 | .055 | -.137 | -1.411 | .165 |
|  | Sex | -.337 | .260 | -.121 | -1.297 | .201 |
|  | Subjective SES | -.062 | .072 | -.080 | -.863 | .392 |
|  | Intercept | 1.282 | .166 | .906 | 7.740 | .000 |
| Meaning in life | (Constant) | 1.171 | 1.945 |  | .602 | .550 |
|  | **Valence** | **-.375** | **.238** | **-.227** | **-1.573** | **.122** |
|  | Age | -.102 | .059 | -.166 | -1.749 | .087 |
|  | Sex | -.071 | .276 | -.023 | -.257 | .798 |
|  | Subjective SES | -.067 | .075 | -.080 | -.898 | .374 |
|  | Intercept | 1.362 | .208 | .940 | 6.556 | .000 |
|  | (Constant) | .903 | 2.340 |  | .386 | .701 |
|  | **Interestedness** | **-.626** | **.285** | **-.385** | **-2.199** | **.033** |
|  | Age | -.126 | .066 | -.205 | -1.924 | .060 |
|  | Sex | -.201 | .313 | -.067 | -.641 | .525 |
|  | Subjective SES | -.091 | .087 | -.108 | -1.042 | .303 |
|  | Intercept | 1.693 | .297 | .985 | 5.691 | .000 |
|  | (Constant) | 1.067 | 1.759 |  | .607 | .547 |
|  | **Activeness** | **-.588** | **.238** | **-.271** | **-2.469** | **.017** |
|  | Age | -.091 | .056 | -.147 | -1.610 | .114 |
|  | Sex | -.196 | .264 | -.065 | -.743 | .461 |
|  | Subjective SES | -.056 | .073 | -.067 | -.769 | .446 |
|  | Intercept | 1.431 | .168 | .936 | 8.505 | .000 |
| Stress | (Constant) | 9.854 | 2.145 |  | 4.595 | .000 |
|  | **Valence** | **.894** | **.263** | **.594** | **3.402** | **.001** |
|  | Age | .055 | .065 | .098 | .856 | .396 |
|  | Sex | .265 | .304 | .097 | .873 | .387 |
|  | Subjective SES | .008 | .082 | .011 | .099 | .921 |
|  | Intercept | -1.356 | .229 | -1.028 | -5.919 | .000 |
|  | (Constant) | 9.859 | 2.426 |  | 4.063 | .000 |
|  | **Interestedness** | **.942** | **.295** | **.636** | **3.189** | **.003** |
|  | Age | .088 | .068 | .156 | 1.290 | .203 |
|  | Sex | .416 | .325 | .151 | 1.281 | .206 |
|  | Subjective SES | .024 | .090 | .031 | .260 | .796 |
|  | Intercept | -1.560 | .308 | -.997 | -5.059 | .000 |
|  | (Constant) | 7.628 | 2.170 |  | 3.516 | .001 |
|  | **Activeness** | **1.064** | **.294** | **.539** | **3.619** | **.001** |
|  | Age | .072 | .069 | .127 | 1.031 | .308 |
|  | Sex | .383 | .326 | .139 | 1.174 | .246 |
|  | Subjective SES | -.021 | .090 | -.027 | -.231 | .818 |
|  | Intercept | -1.053 | .208 | -.757 | -5.073 | .000 |

*Note*. SES, socioeconomic status.

**Supplementary Table 6**. Results of seed-to-voxel functional connectivity associated with FWB measures with *a priori* anatomical search volume (i.e., vmPFC).

| **FWB** | **Seed region** | **Cluster size (*k*)** | **Statistics** | | ***X*** | | ***Y*** | | ***Z*** | |  |
| --- | --- | --- | --- | --- | --- | --- | --- | --- | --- | --- | --- |
|  |  |  | **SVC FWE *p*** | **peak *t*** | |  | |  | |  | |
| MA | NAc R | 99 | <.001 | 4.42 | | -6 | | 38 | | -20 | |
|  | NAc L | 27 | .016 | 3.69 | | -6 | | 38 | | -18 | |
| CMS | NAc R | 212 | <.001 | -6.05 | | -4 | | 40 | | -14 | |
|  | NAc L | 91 | .001 | -4.79 | | -4 | | 38 | | -14 | |

*Note*. CMS, current money management stress; FWB, financial well-being; L, left; MA, material affluence; NAc, nucleus accumbens; R, right; SVC FWE, small volume correction family-wise error; vmPFC, ventromedial prefrontal cortex.

**Supplementary Table 7**. Results of seed-to-voxel functional connectivity associated with FWB measures without *a priori* anatomical search volume for exploratory purposes.

| **FWB** | **Seed region** | **Brain region** | **Cluster size (*k*)** | **Statistics** | | ***X*** | ***Y*** | ***Z*** |
| --- | --- | --- | --- | --- | --- | --- | --- | --- |
|  |  |  |  | **FWE *p*** | **peak *t*** |  |  |  |
| MA | NAc R | vmPFC | 113 | .042 | 4.48 | -6 | 40 | -20 |
|  |  | Insular L | 169 | .006 | 5.45 | -40 | -8 | 0 |
| CMS | NAc R | vmPFC | 310 | <.001 | -6.05 | -4 | 40 | -14 |
|  |  | PostCG L | 127 | .025 | -4.44 | -40 | -32 | 40 |
|  | NAc L | vmPFC | 166 | .009 | -4.79 | -4 | 38 | -14 |
|  |  | IFG/MFG L | 355 | <.001 | -4.42 | -40 | 16 | 38 |
|  |  | SPL L | 118 | .043 | -4.19 | -44 | -40 | 48 |

*Note*. CMS, current money management stress; FWB, financial well-being; IFG, inferior frontal gyrus; L, left; MA, material affluence; MFG, middle frontal gyrus; NAc, nucleus accumbens; PostCG, postcentral gyrus; R, right; SPL, superior parietal lobule; SVC FWE, small volume correction family-wise error; vmPFC, ventromedial prefrontal cortex.

**Supplementary Table 8-1.** Regression results of FWB as the predictor, the affective responses when positive events occurred as the outcome variable, and age, sex, subjective SES, and intercept of each affective state (if applicable) as covariates.

| **Dependent**  **Variables** | **Independent**  **Variables** | **B** | ***SE*** | ***β*** | ***t*** | ***p*** |
| --- | --- | --- | --- | --- | --- | --- |
| Valence | (Constant) | 1.615 | .047 |  | 34.144 | .000 |
|  | **MA** | **.000** | **.001** | **-.038** | **-.539** | **.592** |
|  | Age | .001 | .002 | .042 | .645 | .521 |
|  | Sex | .002 | .007 | .021 | .332 | .741 |
|  | Subjective SES | .001 | .002 | .032 | .467 | .642 |
|  | Intercept | -.049 | .004 | -.837 | -13.703 | .000 |
|  | (Constant) | 1.607 | .047 |  | 34.041 | .000 |
|  | **CMS** | **.000** | **.001** | **.032** | **.484** | **.629** |
|  | Age | .001 | .002 | .040 | .614 | .541 |
|  | Sex | .002 | .007 | .019 | .307 | .760 |
|  | Subjective SES | .001 | .002 | .025 | .395 | .694 |
|  | Intercept | -.049 | .004 | -.840 | -13.916 | .000 |
|  | (Constant) | 1.604 | .048 |  | 33.551 | .000 |
|  | **FFS** | **.001** | **.001** | **.037** | **.581** | **.563** |
|  | Age | .001 | .002 | .052 | .822 | .414 |
|  | Sex | .002 | .007 | .021 | .327 | .744 |
|  | Subjective SES | .000 | .002 | .006 | .101 | .920 |
|  | Intercept | -.050 | .004 | -.855 | -13.927 | .000 |
| Interestedness | (Constant) | .882 | .322 |  | 2.742 | .008 |
|  | **MA** | **-.001** | **.004** | **-.033** | **-.257** | **.798** |
|  | Age | .010 | .012 | .098 | .828 | .410 |
|  | Sex | .044 | .046 | .112 | .968 | .336 |
|  | Subjective SES | .007 | .015 | .061 | .486 | .628 |
|  | Intercept | .048 | .032 | .167 | 1.482 | .142 |
|  | (Constant) | .867 | .321 |  | 2.700 | .008 |
|  | **CMS** | **.000** | **.005** | **.003** | **.021** | **.983** |
|  | Age | .010 | .012 | .104 | .860 | .392 |
|  | Sex | .044 | .046 | .111 | .962 | .339 |
|  | Subjective SES | .006 | .014 | .048 | .403 | .688 |
|  | Intercept | .046 | .032 | .161 | 1.443 | .153 |
|  | (Constant) | .780 | .324 |  | 2.409 | .018 |
|  | **FFS** | **.007** | **.006** | **.134** | **1.154** | **.252** |
|  | Age | .012 | .011 | .123 | 1.050 | .297 |
|  | Sex | .046 | .045 | .117 | 1.017 | .312 |
|  | Subjective SES | .002 | .014 | .013 | .116 | .908 |
|  | Intercept | .038 | .032 | .134 | 1.205 | .232 |
| Activeness | (Constant) | .903 | .379 |  | 2.383 | .020 |
|  | **MA** | **.000** | **.004** | **-.015** | **-.110** | **.913** |
|  | Age | .003 | .014 | .022 | .186 | .853 |
|  | Sex | -.005 | .054 | -.010 | -.085 | .932 |
|  | Subjective SES | .005 | .017 | .035 | .277 | .783 |
|  | Intercept | .051 | .033 | .175 | 1.529 | .130 |
|  | (Constant) | .909 | .380 |  | 2.389 | .019 |
|  | **CMS** | **-.001** | **.006** | **-.022** | **-.180** | **.858** |
|  | Age | .003 | .014 | .030 | .248 | .805 |
|  | Sex | -.005 | .054 | -.010 | -.083 | .934 |
|  | Subjective SES | .003 | .016 | .022 | .187 | .852 |
|  | Intercept | .049 | .033 | .167 | 1.487 | .141 |
|  | (Constant) | .826 | .381 |  | 2.166 | .033 |
|  | **FFS** | **.006** | **.007** | **.106** | **.885** | **.379** |
|  | Age | .004 | .014 | .038 | .321 | .749 |
|  | Sex | -.003 | .054 | -.006 | -.054 | .957 |
|  | Subjective SES | .000 | .016 | .002 | .018 | .986 |
|  | Intercept | .042 | .033 | .144 | 1.263 | .210 |

*Note*. CMS, current money management stress; FFS, future financial security; MA, material affluence; SES, socioeconomic status.

**Supplementary Table 8-2.** Regression results of FWB as the predictor, the affective responses when negative events occurred as the outcome variable, and age, sex, subjective SES, and intercept of each affective state (if applicable) as covariates.

| **Dependent**  **Variables** | **Independent**  **Variables** | **B** | ***SE*** | ***β*** | ***t*** | ***p*** |
| --- | --- | --- | --- | --- | --- | --- |
| Valence | (Constant) | .012 | .830 |  | .014 | .989 |
|  | **MA** | **-.008** | **.009** | **-.112** | **-.909** | **.366** |
|  | Age | .042 | .030 | .156 | 1.391 | .168 |
|  | Sex | .349 | .119 | .320 | 2.937 | .004 |
|  | Subjective SES | .045 | .038 | .138 | 1.169 | .246 |
|  | Intercept | .118 | .059 | .212 | 1.991 | .050 |
|  | (Constant) | -.195 | .833 |  | -.234 | .815 |
|  | **CMS** | **.008** | **.013** | **.067** | **.576** | **.566** |
|  | Age | .042 | .031 | .158 | 1.386 | .169 |
|  | Sex | .345 | .119 | .316 | 2.893 | .005 |
|  | Subjective SES | .036 | .036 | .111 | .992 | .324 |
|  | Intercept | .111 | .059 | .200 | 1.895 | .062 |
|  | (Constant) | .173 | .833 |  | .208 | .836 |
|  | **FFS** | **-.023** | **.015** | **-.168** | **-1.522** | **.132** |
|  | Age | .041 | .030 | .154 | 1.398 | .166 |
|  | Sex | .340 | .118 | .311 | 2.888 | .005 |
|  | Subjective SES | .043 | .035 | .134 | 1.228 | .223 |
|  | Intercept | .126 | .058 | .227 | 2.153 | .034 |
| Interestedness | (Constant) | -2.252 | .665 |  | -3.386 | .001 |
|  | **MA** | **-.003** | **.007** | **-.033** | **-.340** | **.735** |
|  | Age | .030 | .024 | .108 | 1.235 | .221 |
|  | Sex | .117 | .095 | .104 | 1.225 | .224 |
|  | Subjective SES | .037 | .031 | .111 | 1.200 | .234 |
|  | Intercept | .461 | .058 | .668 | 7.992 | .000 |
|  | (Constant) | -2.262 | .667 |  | -3.393 | .001 |
|  | **CMS** | **-.002** | **.011** | **-.021** | **-.229** | **.819** |
|  | Age | .033 | .024 | .119 | 1.336 | .185 |
|  | Sex | .117 | .095 | .105 | 1.226 | .224 |
|  | Subjective SES | .030 | .029 | .091 | 1.043 | .300 |
|  | Intercept | .454 | .057 | .657 | 7.933 | .000 |
|  | (Constant) | -2.228 | .675 |  | -3.303 | .001 |
|  | **FFS** | **-.005** | **.012** | **-.033** | **-.384** | **.702** |
|  | Age | .030 | .024 | .109 | 1.261 | .211 |
|  | Sex | .115 | .095 | .103 | 1.202 | .233 |
|  | Subjective SES | .035 | .029 | .105 | 1.227 | .224 |
|  | Intercept | .461 | .057 | .668 | 8.056 | .000 |
| Activeness | (Constant) | -3.319 | .930 |  | -3.568 | .001 |
|  | **MA** | **-.016** | **.010** | **-.140** | **-1.499** | **.138** |
|  | Age | .042 | .034 | .104 | 1.234 | .221 |
|  | Sex | .089 | .134 | .055 | .669 | .505 |
|  | Subjective SES | .067 | .043 | .139 | 1.561 | .123 |
|  | Intercept | .642 | .072 | .727 | 8.947 | .000 |
|  | (Constant) | -3.557 | .948 |  | -3.753 | .000 |
|  | **CMS** | **.002** | **.015** | **.009** | **.103** | **.918** |
|  | Age | .050 | .035 | .125 | 1.434 | .155 |
|  | Sex | .085 | .136 | .052 | .627 | .532 |
|  | Subjective SES | .041 | .041 | .084 | .980 | .330 |
|  | Intercept | .614 | .072 | .695 | 8.532 | .000 |
|  | (Constant) | -3.231 | .941 |  | -3.432 | .001 |
|  | **FFS** | **-.026** | **.018** | **-.127** | **-1.497** | **.138** |
|  | Age | .045 | .034 | .112 | 1.333 | .186 |
|  | Sex | .077 | .134 | .048 | .578 | .565 |
|  | Subjective SES | .055 | .040 | .113 | 1.359 | .178 |
|  | Intercept | .641 | .072 | .726 | 8.952 | .000 |

*Note*. CMS, current money management stress; FFS, future financial security; MA, material affluence; SES, socioeconomic status.


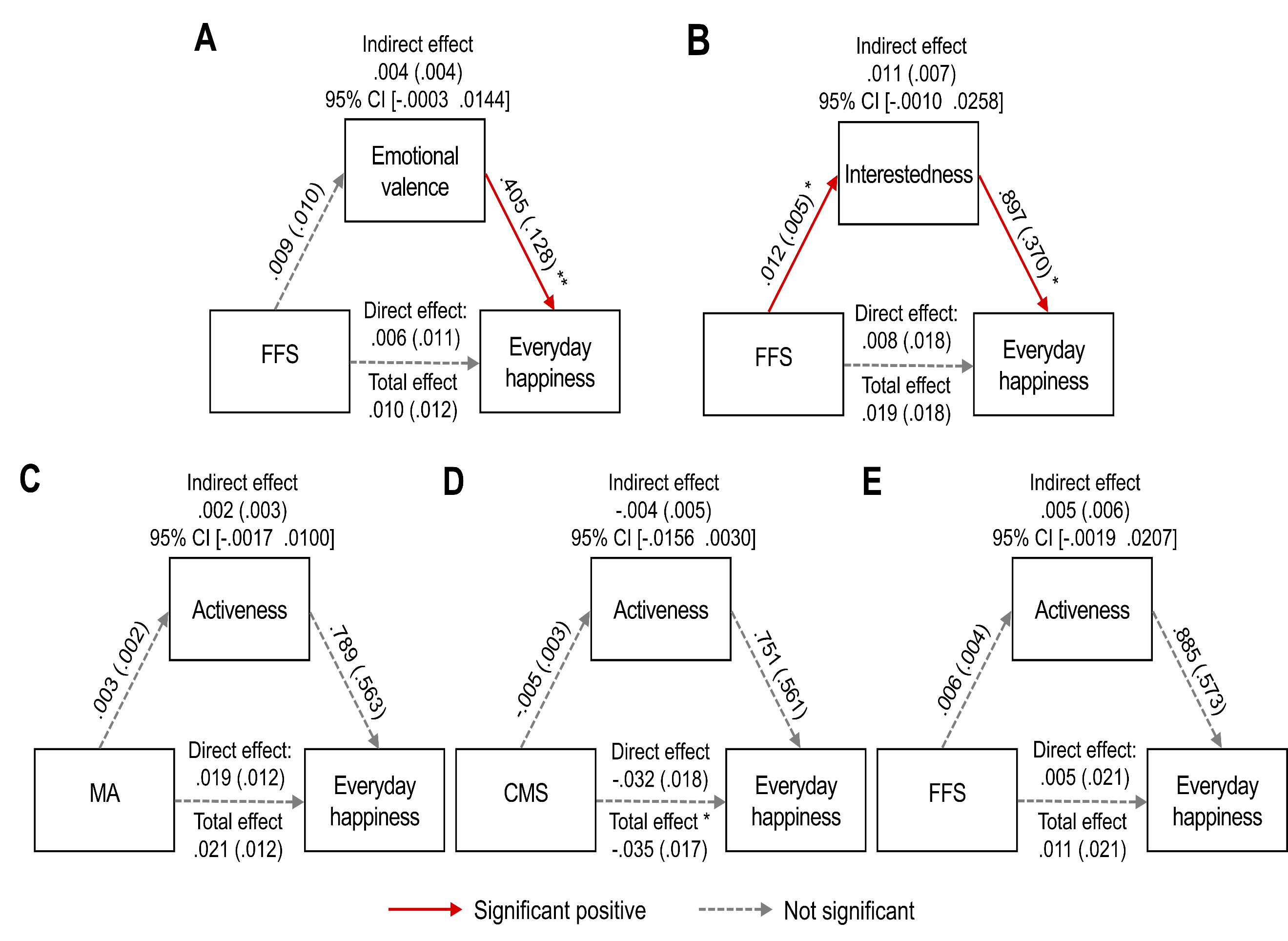


**Supplementary Figure 1.** The mediation results when entering FFS as the predictor, the anticipatory responses in emotional valence or interestedness during positive expectations as the mediator, and the composite score of everyday happiness as the outcome variable (**A** and **B**). The mediation results when entering FWB as the predictor, the anticipatory responses in emotional valence, interestedness, or activeness during positive expectations as the mediator, and the composite score of everyday happiness as the outcome variable (**C**, **D**, and **E**). Age, sex, subjective SES, and intercept of each affective state as covariates. Path coefficients are listed for each path, with standard errors in parentheses.

*Note*. CMS, current money management stress; FFS, future financial security; MA, material affluence; FWB, financial well-being. **p* < 0.05, ***p* < 0.01.


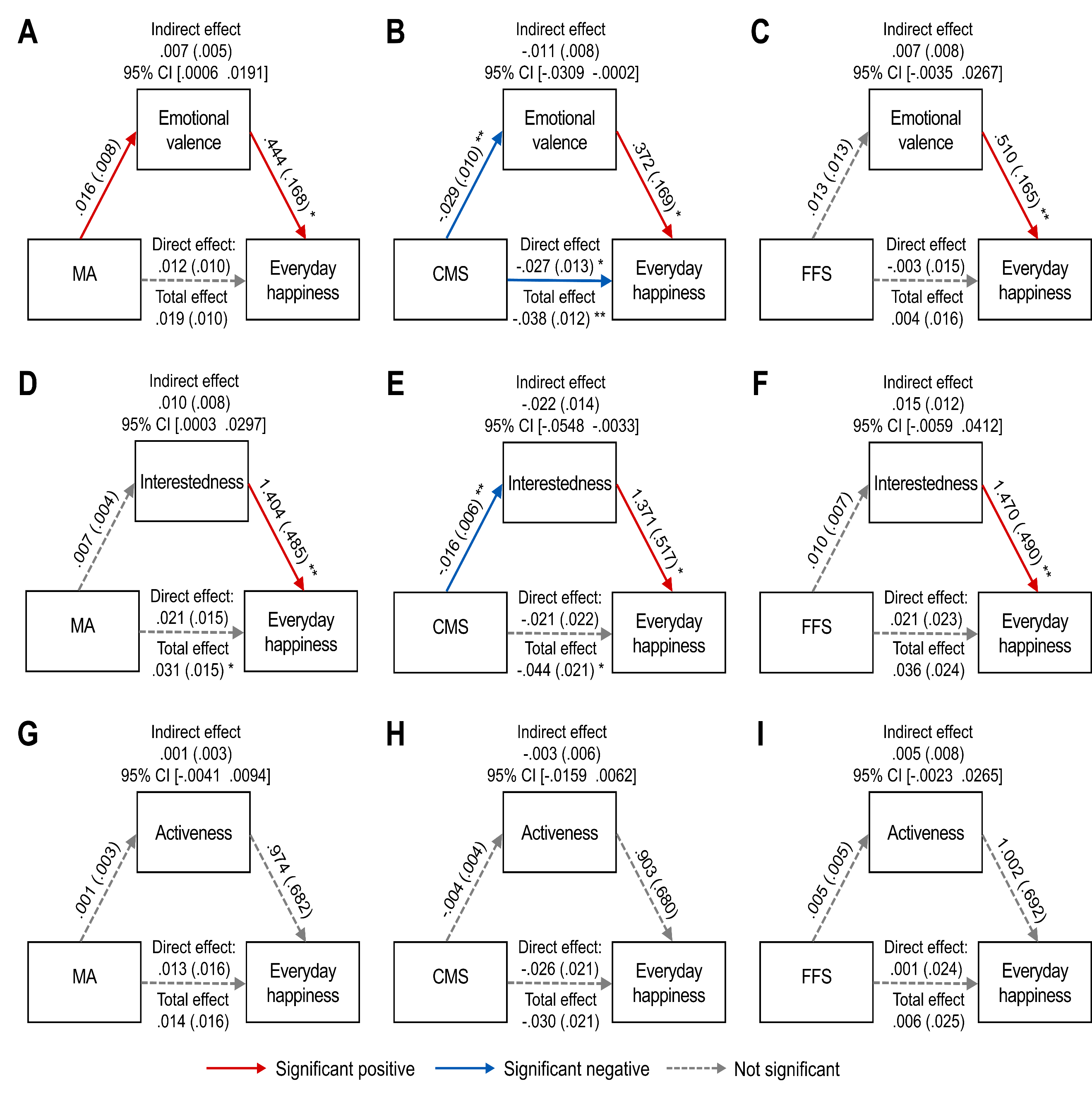


**Supplementary Figure 1*.** The mediation results when entering FWB as the predictor, the anticipatory responses in emotional valence, interestedness or, activeness during positive expectations as the mediator, and the composite score of everyday happiness as the outcome variable (*N* = 54). Age, sex, subjective SES, and intercept of each affective state as covariates. Path coefficients are listed for each path, with standard errors in parentheses.

*Note*. CMS, current money management stress; FFS, future financial security; MA, material affluence; FWB, financial well-being. **p* < 0.05, ***p* < 0.01.


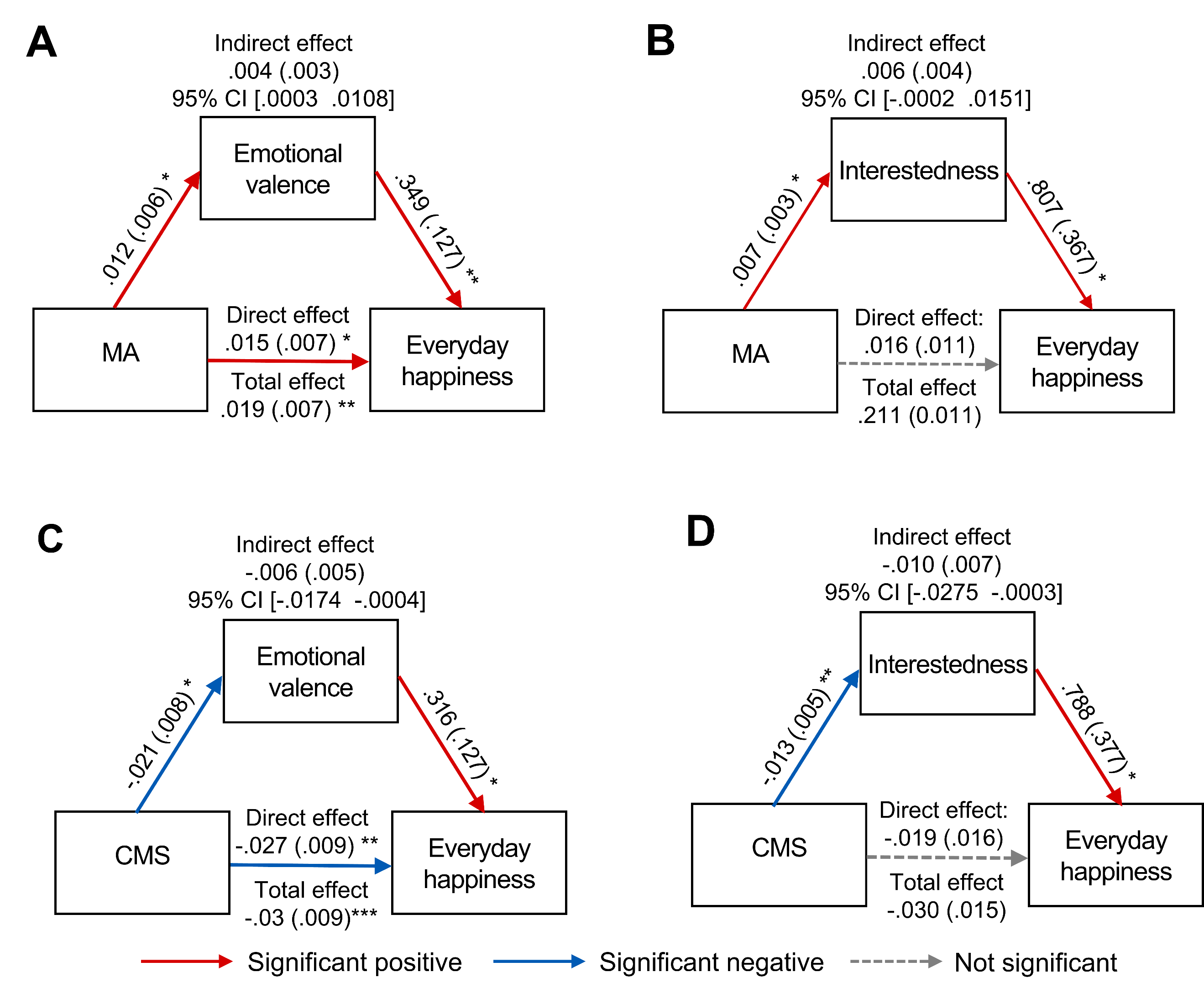


**Supplementary Figure 1†.** The mediation results when entering FWB as the predictor, the anticipatory responses in emotional valence and interestedness during positive expectations as the mediator, and the composite score of everyday happiness as the outcome variable. Age, sex, subjective SES, optimism, and intercept of each affective state as covariates. Path coefficients are listed for each path, with standard errors in parentheses.

*Note*. CMS, current money management stress; MA, material affluence; FWB, financial well-being. **p* < 0.05, ***p* < 0.01, ****p* < 0.001.


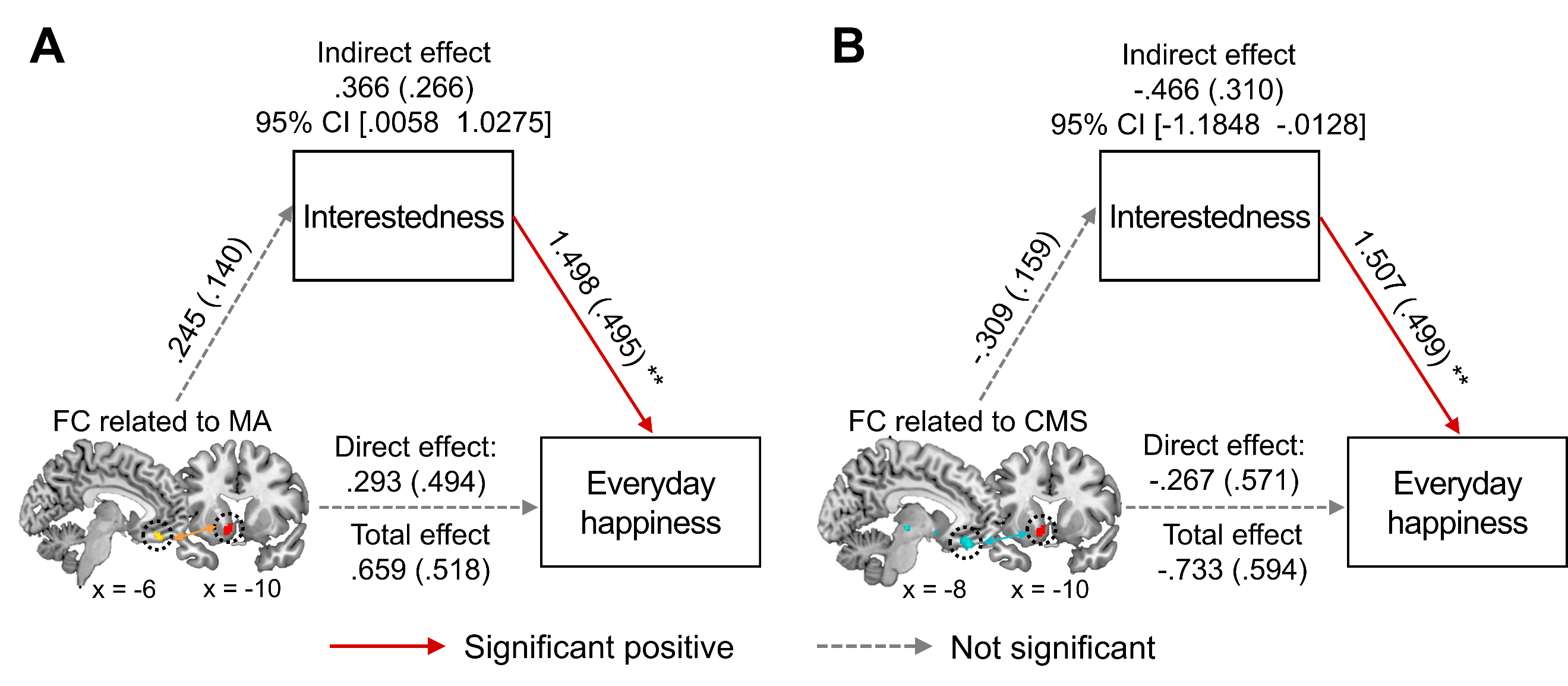


**Supplementary Figure 2.** The mediation results when entering the FCs between the left NAc and the vmPFC associated with MA (**A**) and CMS (**B**) as the predictor, the anticipatory responses in interestedness during positive expectations as the mediator, and the composite score of everyday happiness as the outcome variable. Age, sex, subjective SES, and intercept of each affective state as covariates. Path coefficients are listed for each path, with standard errors in parentheses.

*Note*. FC, functional connectivity; CMS, current money management stress; MA, material affluence; NAc, nucleus accumbens; vmPFC, ventromedial prefrontal cortex. ***p* < 0.01.


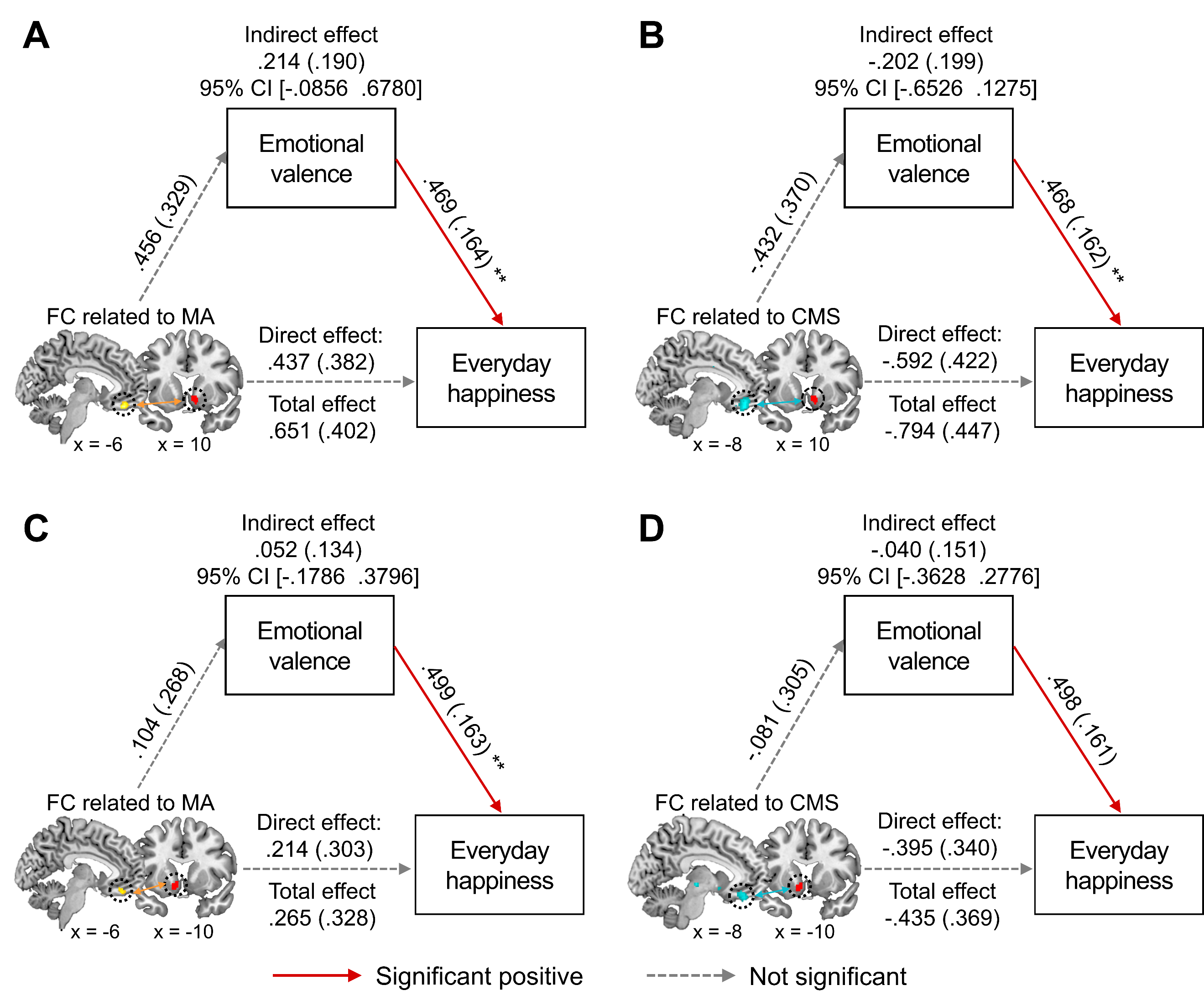
 **Supplementary Figure 3.** The mediation results when entering the FCs between the right NAc and the vmPFC associated with MA (**A**) and CMS (**B**) as the predictor, the anticipatory responses in emotional valence during positive expectations as the mediator, and the composite score of everyday happiness as the outcome variable. The mediation results when entering the FCs between the left NAc and the vmPFC associated with MA (**C**) and CMS (**D**) with the same mediator and outcome variable. Age, sex, subjective SES, and intercept of each affective state as covariates. Path coefficients are listed for each path, with standard errors in parentheses.

*Note*. FC, functional connectivity; CMS, current money management stress; MA, material affluence; NAc, nucleus accumbens; vmPFC, ventromedial prefrontal cortex. ***p* < 0.01.


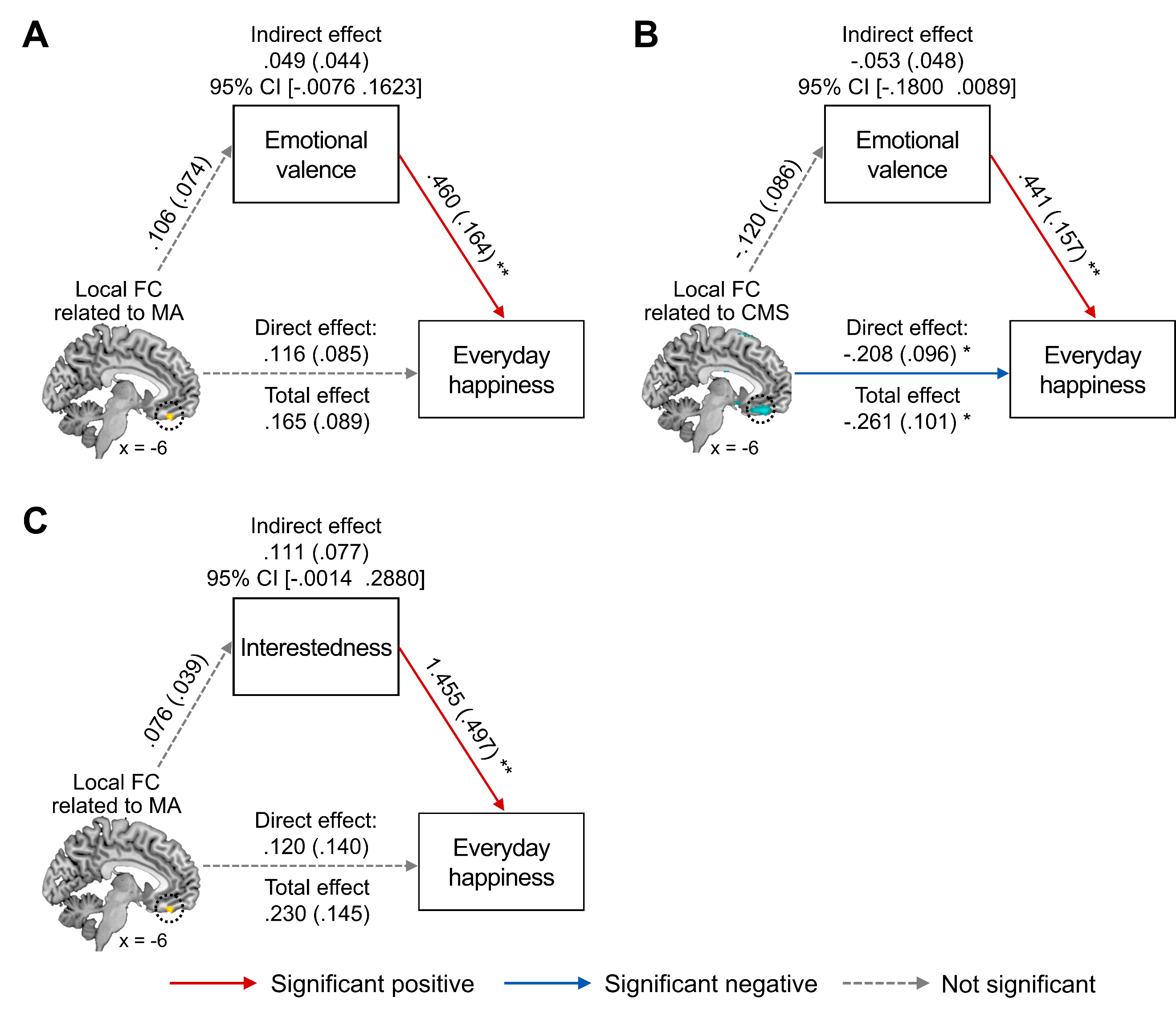


**Supplementary Figure 4.** The mediation results when entering the local FCs within the vmPFC associated with MA (**A**) and CMS (**B**) as the predictor, the anticipatory responses in emotional valence during positive expectations as the mediator, and the composite score of everyday happiness as the outcome variable. The mediation result when entering the local FC associated with MA as the predictor and the anticipatory responses in interestedness as the mediator with the same outcome variable (**C**). Age, sex, subjective SES, and intercept of each affective state as covariates. Path coefficients are listed for each path, with standard errors in parentheses.

*Note*. FC, functional connectivity; CMS, current money management stress; MA, material affluence; vmPFC, ventromedial prefrontal cortex. ***p* < 0.01.
